# Impact of environmental factors on neglected emerging arboviral diseases

**DOI:** 10.1371/journal.pntd.0005959

**Published:** 2017-09-27

**Authors:** Camila Lorenz, Thiago S. Azevedo, Flávia Virginio, Breno S. Aguiar, Francisco Chiaravalloti-Neto, Lincoln Suesdek

**Affiliations:** 1 Department of Parasitology, Instituto Butantan, Sao Paulo, Sao Paulo, Brazil; 2 Department of Geography, Universidade Estadual Paulista, Rio Claro, Sao Paulo, Brazil; 3 Department of Epidemiology, School of Public Health, Universidade de São Paulo, Sao Paulo, Brazil; 4 Instituto de Medicina Tropical, Sao Paulo, São Paulo, Brazil; Fundacao Oswaldo Cruz, BRAZIL

## Abstract

**Background:**

Brazil is a tropical country that is largely covered by rainforests and other natural ecosystems, which provide ideal conditions for the existence of many arboviruses. However, few analyses have examined the associations between environmental factors and arboviral diseases. Thus, based on the hypothesis of correlation between environment and epidemiology, the proposals of this study were (1) to obtain the probability of occurrence of Oropouche, Mayaro, Saint Louis and Rocio fevers in Brazil based on environmental conditions corresponding to the periods of occurrence of the outbreaks; (2) to describe the macroclimatic scenario in Brazil in the last 50 years, evaluating if there was any detectable tendency to increase temperatures and (3) to model future expansion of those arboviruses in Brazil based on future temperature projections.

**Methodology/Principal findings:**

Our model assessed seven environmental factors (annual rainfall, annual temperature, elevation, seasonality of temperature, seasonality of precipitation, thermal amplitude, and daytime temperature variation) for their association with the occurrence of outbreaks in the last 50 years. Our results suggest that various environmental factors distinctly influence the distribution of each arbovirus, with temperature being the central determinant of disease distribution in all high-risk areas. These areas are subject to change, since the average temperature of some areas has increased significantly over the time.

**Conclusions/Significance:**

This is the first spatio-temporal study of the Oropouche, Mayaro, Saint Louis, and Rocio arboviruses, and our results indicate that they may become increasingly important public health problems in Brazil. Thus, next studies and control programs should include these diseases and also take into consideration key environmental elements.

## Author summary

The Oropouche, Mayaro, Saint Louis, and Rocio viruses are neglected emerging mosquito-borne viruses that are spreading and causing wide-scale epidemics in South America. However, under-reporting of these cases is possible, as the symptoms are shared with other endemic diseases. Moreover, little is known regarding environmental conditions that favor these tropical outbreaks of arboviral diseases. This study examined the association of environmental factors with the probability of occurrence of Oropouche, Mayaro, Saint Louis and Rocio fever outbreaks (present and future) and finds that temperature is a central variable that determines the distribution of high-risk areas. This fact is very worrying, because the average temperature of some areas has increased significantly over the time. Results from this study strongly suggest that these four diseases have the potential to become important public health problems or become increasingly relevant in Brazil and other tropical areas in the coming years and should be monitored as part of effective control programs.

## Introduction

Arboviruses have become important and constant threats in tropical regions, due to rapid climate change, deforestation, population migration, disorderly occupation of the urban areas, and precarious sanitary conditions that favor viral amplification and transmission [1]. Climate fluctuations produce conditions that accelerate arbovirus epidemics, directly affecting global public health [2]. Abnormally high temperatures for example, affect populations of insect vectors, and arboviral diseases, by influencing: the survival and replication of the virus, susceptibility of the vector to viruses, distribution of vectors, extrinsic incubation period of a virus in the insect, and seasonality of virus transmission patterns [3,4]. Besides that, arboviruses are highly spreadable because their vectors can be carried long distances, and even between countries or continents, which can lead to pandemics.

Brazil is the largest South American country and has a population of approximately 207 million in an area of 8,514,215 km^2^ [5]. More than >30% of Brazil remains covered by rainforests and other natural ecosystems, despite the high rate of deforestation [5]. These natural environments can harbor many arboviruses that are maintained in different zoonotic cycles. For example, approximately 200 different arbovirus species have been isolated in Brazil, including 40 species that can cause human diseases [6,7]. Although it is acknowledged that dengue, zika, chikungunya, and recently, yellow fever, are today the most important emerging and re-emerging arboviral diseases in Brazil, in this study we focused on others that have been neglected and consequently, are less discussed in medical literature. These include Oropouche (ORO), Mayaro (MAY), Saint Louis (SLE), and Rocio (ROC). Besides the lack of specific tests to identify these diseases, the similarities among the symptoms are very high; fever, for example, is common to all of them. This makes the correct diagnosis very difficult and in most cases may have been underreported.

### Oropouche (ORO)

The Oropouche virus (Orthobunyavirus genus) was first isolated in 1955 from a febrile human patient and *Coquillettidia venezuelensis* mosquitoes in Trinidad and Tobago [8]. Five years later, the virus was detected in a Brazilian territory in a sloth (*Bradypus tridactylus*) and in *Ochlerotatus serratus* mosquitoes [9]. Since then, ORO has been a common cause of explosive urban epidemics in the Amazon region, affecting large cities such as Belem and Manaus.

This virus is transmitted among vertebrate hosts, such as marsupials, sloths, primates, and birds, through a generally wild transmission cycle by the *Ochlerotatus serratus* and *Culex quinquefasciatus* mosquitoes. Notably, this arbovirus has adjusted to an urban transmission cycle with humans as the main reservoir and *Culicoides paraensis* (Ceratopogonidae) as the main vector. Thus, there is a worrisome risk of ORO emergence in the densely populated coast of Brazil, which cover the northeastern and southeastern regions, considering that vector *C*. *paraensis* is present in low-altitude areas of the entire Brazilian territory [7,10]. Moreover, *Cx*. *quinquefasciatus* mosquitoes are spread throughout the Brazilian cities, suggesting the need to pay more attention to this mosquito species too.

ORO is one of the most important arboviral diseases in the Americas, especially in the Brazilian Amazon region. However, because ORO fever is not considered a reportable disease, it is difficult to estimate its incidence during outbreaks, although serological surveys are useful in this setting. Thus, research has indicated that approximately 500,000 people in the Amazon region may have been infected with the ORO virus since the early 1960s [6].

Most epidemics of ORO fever typically occur during the rainy season. However, some epidemics have also extended into the dry season, although with less intensity. The seasonal nature of the ORO is most likely linked to the higher density of the populations of the vector *C*. *paraensis* in months with higher levels of rainfall, combined with a higher concentration of exposed hosts. Unfortunately, the diagnosis of ORO can be confused with other acute febrile diseases that are endemic in the Amazon region, such as malaria and dengue [11].

### Mayaro (MAY)

The MAY virus, belonging to the Alphavirus genus, has been responsible for outbreaks of acute febrile illness and arthralgia syndrome in northern and midwestern Brazil, as well as Peru, Bolivia, and Venezuela [7,12]. This virus was first detected and isolated in 1954, from rural workers in Trinidad [13].

Human cases of MAY are sporadic and mainly involve people who live in rainforests, as the main vector is the *Haemagogus* mosquitoes that are common in those forests. Vertebrate hosts are mainly mammals, although there is some evidence of bird infections in southern Brazil. *Aedes* mosquitoes can also transmit the virus in rural, suburban, and urban areas [14]. The course of 3–5 day of illness is characterized by fever, headache, myalgia, rash, and pain, mainly in the large joints, and less often, arthritis [15,16]. The spread of this virus can extend to cities through an infected human or through birds that can travel long distances in a short time, and adapt to a new cycle that involves humans as reservoirs.

This febrile illness occurs throughout the year, more frequently in the rainy season, as with dengue and ORO, and affects people of both sexes, of all ages. The estimated transmission of the virus in Manaus, state of Amazonas, is about 2 million people. This is a public health problem because there is no vaccine, and vector control is not feasible [12].

### Saint Louis Encephalitis (SLE)

The SLE virus belongs to the Japanese encephalitis virus complex, which is within the *Flavivirus* genus, and Flaviviridae family [17]. The virus was first isolated in 1933from suspensions of human intracerebral brain samples that had been inoculated postmortem with tissues from rhesus monkeys and white mice (Saint Louis, Missouri, USA) [18]. Currently, the SLE virus is broadly distributed throughout all Americas (from Canada to Argentina), and has neurotropic characteristics [12]. It causes an acute disease in humans, with manifestations that range from febrile syndrome to fatal meningoencephalitis [19]. Reports of fatal cases vary from 5% to 20%; however, the numbers are even higher among the elderly population [20].

Transmission of the SLE virus occurs through *Culex* mosquitoes, and migratory birds spread the virus and other forms of encephalitis along their migratory routes [12]. Despite rare cases of the isolation of SLE virus in humans in Brazil, the antibodies of this virus were found in approximately 5% of the populations of the Northern and Southeastern Regions [12]. Recently, there was an outbreak of SLE in the country, which occurred simultaneously with that of dengue in São José do Rio Preto (Sao Paulo) [21]. During this outbreak, some patients with SLE exhibited hemorrhagic manifestations, such as a positive tourniquet test, petechiae, and bleeding [21].

### Rocio (ROC)

The ROC virus was first isolated in 1975 from a fatal case of encephalitis in a restricted area of the Atlantic Forest (Ribeira River Valley Sao Paulo) [7]. The case was detected during the 1973–1980 outbreak which caused an estimated 1,000 cases of encephalitis in more than 20 municipalities. The mortality rate was 10%, and among the survivors, about 200 suffered balance or mobility sequelae [7]. It is unclear how the ROC virus spread to this region and why it subsequently disappeared 7 years later, although antibodies have been detected in rural residents of southeastern and northeastern Brazil. [22,23]. Based on the viral isolation and serological data, it is believed that the ROC virus is maintained in a transmission cycle that involves wild birds, including some migratory species, as the reservoirs, and *Aedes* and *Psorophora* mosquitoes, as the vectors.

Despite the availability of a comprehensive record in the literature for these relevant diseases, to the best of our knowledge, no predictive models have been developed in this context. In this study, we analyze and illustrate how these four mosquito-borne diseases can have serious public health implications, or increase their relevance in the future. Thus, the study’s goals were: (a) to obtain the probability of occurrence of ORO, MAY, ROC and SLE in Brazil, based on environmental conditions corresponding to the periods of occurrence of the outbreaks; (b) to describe the macroclimatic scenario in Brazil in the last 50 years, evaluating any detectable tendency to increase temperatures and (c) to predict future expansion of ORO, MAY, SLE and ROC in Brazil, based on future temperature projections for 2046–2065 and 2071–2100, using two different scenarios of greenhouse gas emissions.

## Methods

### Study area and data source

The approximate locations of human ORO, MAY, SLE, and ROC cases were determined using sites that were identified in the literature between 1961 and 2012 (Table 1). Data were exhaustively collected using searches of the PubMed and Google Scholar databases (search term: “Oropouche” OR “Mayaro” OR “Saint Louis” OR “Rocio” AND “Brasil” OR “Brazil”) and the library of University of Sao Paulo, Brazil. We have included all records of diseases in Brazilian municipalities reported in epidemiological bulletins since the very first record up until 2012. The criterion for inclusion of a municipality in the analysis was presence ≥ 1 of ORO, MAY, SLE or ROC case. This is because the World Health Organization has stated “*a single case of a communicable disease long absent from a population*, *or caused by an agent (e*.*g*. *bacterium or virus) not previously recognized in that community or area*, *or the emergence of a previously unknown disease*, *may also constitute an outbreak*” [24].

**Table 1:** Brazilian municipalities that have presented arbovirus outbreaks in the 1961–2012 interim. (Data from Google Scholar and Pubmed databases. Searching topics = “Oropouche” OR “Mayaro” OR “Saint Louis” OR “Rocio” AND “Brasil” OR “Brazil”). The acronyms next to each municipality indicate the State.

Virus	Municipality	Epidemic Year	References
**Oropouche**	Belém (PA)	1961, 1968, 1979, 1980	[25–29]
	Bragança (PA)	1967, 1979–1980	
	Baião (PA)	1972	
	Santarém region (PA)	1974–1975	
	Itupiranga (PA)	1975	
	Tomé Açu (PA)	1978	
	Portel (PA)	1979	
	Bragantina region (PA)	1979–1980, 2006	
			
	Mazagão (PA)	1980	
	Barcelos (AM)	1980	
	Manaus (AM)	1980–1981	
	Tocantinópolis (TO)	1988	
	Porto Franco (MA)	1988	
	Ouro Preto d'Oeste (RO)	1991	
	Ariquemes (RO)	1991	
	Serra Pelada (PA)	1994	
	Brasil Novo (PA)	1996	
	Novo Airão (AM)	1996	
	Oriximiná (PA)	1996	
	Vitória do Xingu (PA)	1996	
	Xapuri (AC)	1996	
	Parauapebas (PA)	2003	
	Porto de Moz (PA)	2004	
**Mayaro**	Belterra (PA)	1977–1978	[30–36]
	Conceição do Araguaia (PA)	1981	
	Itaruma (GO)	1987	
	Benevides (PA)	1991	
	Peixe (TO)	1991	
	Acrelândia (AC)	2004	
	Manaus (AM)	2007–2008	
	Santa Bárbara (PA)	2008	
	Sinop (MT)	2011–2012	
	Cuiabá (MT)	2012	
	Sorriso (MT)	2012	
	Várzea Grande (MT)	2012	
	Nossa Senhora do Livramento (MT)	2012	
**Saint Louis**	São Pedro (SP)	2004	[37–39]
	Ribeirão Preto (SP)	2006	
	São José do Rio Preto (SP)	2007	
**Rocio**	Cubatão (SP)	1975	[40–43]
	Guarujá (SP)	1975	
	Itanhaém (SP)	1975	
	São Vicente (SP)	1975	
	Mongaguá (SP)	1975	
	Praia Grande (SP)	1975	
	Santos (SP)	1975	
	Cananéia (SP)	1975–1976	
	Iguape (SP)	1975–1976	
	Itariri (SP)	1975–1976	
	Jacupiranga (SP)	1975–1976	
	Juquiá (SP)	1975–1976	
	Miracatu (SP)	1975–1976	
	Pariquera-Açu (SP)	1975–1976	
	Pedro de Toledo (SP)	1975–1976	
	Peruíbe (SP)	1975–1976	
	Registro (SP)	1975–1976	
	Sete Barras (SP)	1975–1976	
	Barra do Turvo (SP)	1976	
	Eldorado Paulista (SP)	1976	

To determine the ecological and climatic conditions associated with ORO, MAY, SLE, and ROC mosquito-borne disease outbreaks, we examined the relationships between the locations of the outbreaks and seven variables: annual rainfall (RAIN, mm), annual temperature (TEMP, °C), elevation (ELEV, m), seasonality of temperature (SEA-TEMP), seasonality of precipitation (SEA-RAIN), thermal amplitude (THER-AMP), and daytime temperature variation (DTV). The SEA-TEMP value was calculated as the standard deviation of the average monthly temperatures. The THERM-AMP value was calculated by subtracting the minimum temperature during the coldest month from the maximum temperature during the hottest month. The SEA-RAIN value was calculated as the coefficient of variation for average monthly precipitation. The mean DTV value was calculated by subtracting the mean minimum temperature from the mean maximum temperature. All weather data were obtained in ASCII-raster format files and using the "LAT/LONG" geodetic coordinate system (Datum WGS-84). These data were obtained from the WorldClim—Global Climate Data database, which contains representative observational data for 1950–2000 that were interpolated to a resolution of 30 arc-seconds (approximately 1 km). As the environmental variables were expressed in various units, the principal components analysis (PCA) was performed after standardizing the variables using a Pearson correlation matrix. The temperature layers for 1970–2010 were obtained from the National Institute of Meteorology [44], and data regarding other variables were obtained from the AMBDATA [45] and WorldClim [46] databases.

### Data analysis

Our analysis included all probable (clinically diagnosed) and confirmed (serological) cases of persons with the onset of the disease from 1961 through to 2012. For each disease, the environmental variables analyzed were those that corresponded with the years of outbreaks: ORO (between 1961 and 2006), MAY (between 1977 and 2012), SLE (between 2004 and 2007) and ROC (1975 and 1976). The database was developed based on the presence and absence of arboviroses. We considered value **1** for years with at least one case (or more) of ORO, MAY, SLE or ROC, and value **zero** for other years (no occurrence), during the period studied (1961 to 2012). Table 1 shows the municipalities that had cases of these arboviruses and the years in which they occurred.

The PCA was performed using R software to preselect the environmental variables that had the greatest influence on the distributions of each disease [47]. The PCA approach was used for two reasons. First, PCA facilitates the identification and elimination of covariant variables, which is a key procedure for avoiding analytical artefacts. Second, PCA has been widely used in equivalent studies and then facilitates comparisons, reproducibility, and future meta-analysis. After the PCA, we selected the four most representative eigenvectors of the variables for each disease, which were used for Maxent analysis (version 3.3.3 k: a machine learning algorithm for modeling species distributions based on existing data and environmental variables) [48,49]. The data selection was performed according to the criterion of maximum entropy, with the original variables that reached maximum and minimum values within the ordered ranking of each principal component, because they describe the full range of data variation. The Maxent model may be expressed as:

$$p[f_{j}]=\frac{1}{N}\sum_{i=1}^{N}f_{i}(x_{i})$$

Where: x^*^ = the geographical region of interest; x = {x_1_,x_2_ …, x_N_} with x ∈ x^*^; x → observed points at x^*^; f_j_ = f_1´_, f_2´_ …,f_m_ (environmental variables); N = the number of observed cases; and p = the probability of disease occurrence. The model was run 25 times, while withholding a difference of 10% of the localities for each run to estimate the parameters and its precision. The potential distribution maps were created by interpolating the occurrence points and the similarity measures of the environmental variables in each pixel (i.e., a known observation probability value can be assigned to each pixel by calculating a probability whose exponent is a quadratic function). To describe the temperature change patterns in Brazil during the sampled 50 years, we used the kriging method [50, 51] and data from approximately 250 monitoring stations throughout Brazil. This approach generated a map by estimating the value at each node of a regular grid, which was superimposed over the area of interest, and then a contouring program was applied to draw iso-level curves. We used a 250 × 250 grid of Brazil map, which provides 62,500 sections, because it was the maximum map resolution with a minimum required amount of computational time. R software [47] was also used to evaluate the temporal trend in temperature during the last five decades.

Future climate data were integrated using two global climate models (GCMs): the HadGEM2-ES [52] and MIROC-5 [53], which were selected for their different strengths. The HadGEM2-ES model is a stable model that represents a realistic state of the climate, vegetation, and oceanic biology, without the need for artificial corrections. On the other hand, the MIROC-5 model also includes components of the Earth’s system and climate change, in relation to anthropogenic radiation. The advantage of using this model is that it increases the accuracy of short-term climate prediction, as it can be affected by both anthropogenic and intrinsic fluctuations of the climate system. The spatial resolution of the GCMs was the same as that of the environmental variables (30 arc-seconds, approximately 1 km). The comparison method was the same as for the Maxent model, although the probability calculation for the GCMs incorporated a comparison of the present and future environmental conditions. To obtain future climate scenarios using GCMs, it is also necessary to choose a condition for evolution of the greenhouse gas emissions (GGE), during the period when the future climate is projected. In our prediction we used two different scenarios: low emission (RCP 2.6) and very high emission (RCP 8.5), detailed in the Special Report on Emissions Scenarios by the Intergovernmental Panel on Climate Change [54]. In the first case, the global temperature tends to increase by 1.0°C and can reach a temperature anomaly ranging from 0.4 to 1.6°C and 0.3 to 1.7°C between 2046–2065 and 2081–2100, respectively [55]. In the second scenario, with high GGE, the global temperature tends to increase 2.0 to 3.7°C and can reach to a thermal anomaly ranging from 1.4 to 2.6°C and 2.6 to 4.8°C between 2046–2065 and 2081–2100, respectively [56,57].

The models of future expansion of ORO, MAY, SLE and ROC in Brazil were then projected into the timeline, and the two future climatic conditions (2046–2065 and 2071–2100), to identify areas suitable for those diseases. A map of raw temperature projections from the GCMs used to drive the disease models can be seen at S1 Fig. The default Maxent auto feature setting was used (linear, quadratic, product, threshold, and hinge). The maps were edited using QGis software 2.10.1.

## Results

**Fig 1:**
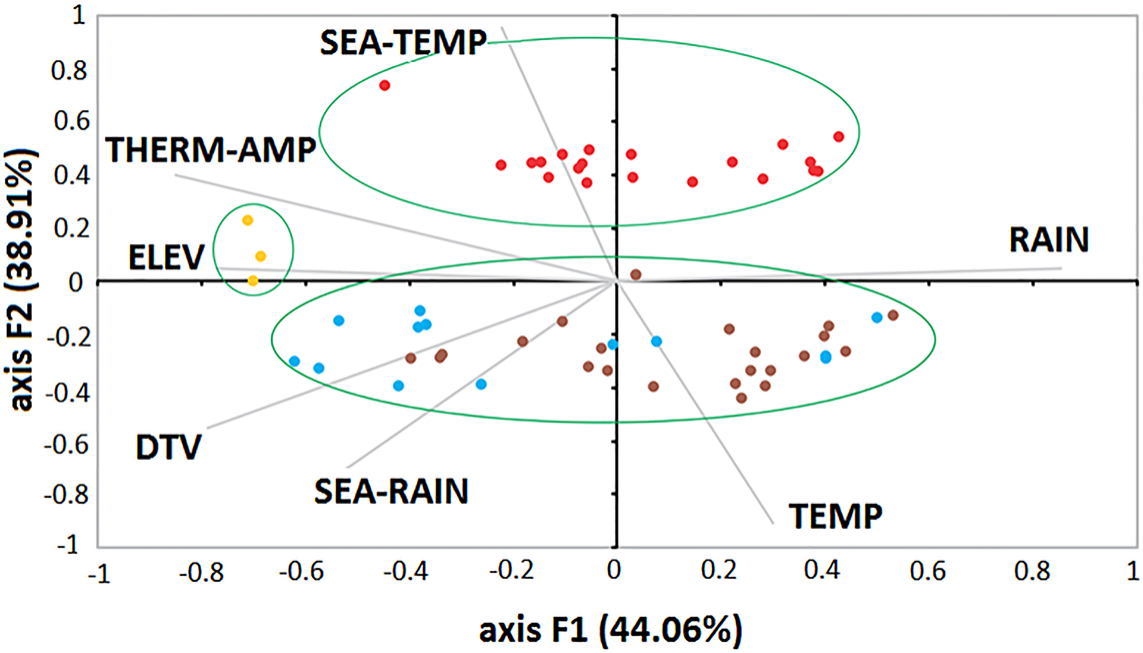
PCA of arboviruses. This PCA showing the distribution of ORO (brown), MAY (blue), SLE (yellow) and ROC (red) cases according to environmental variables. The green ellipses show the main clusters: ROC, SLE and ORO+MAY.

Through PCA of climatic factors, it was possible to identify three main groups: ROC, SLE and ORO + MAY (Fig 1). It is important to note that both diseases ORO and MAY occurred more in the North and Midwest of the country. The first two components (F1 and F2) were able to explain 82.96% of the variation.

**Fig 2:**
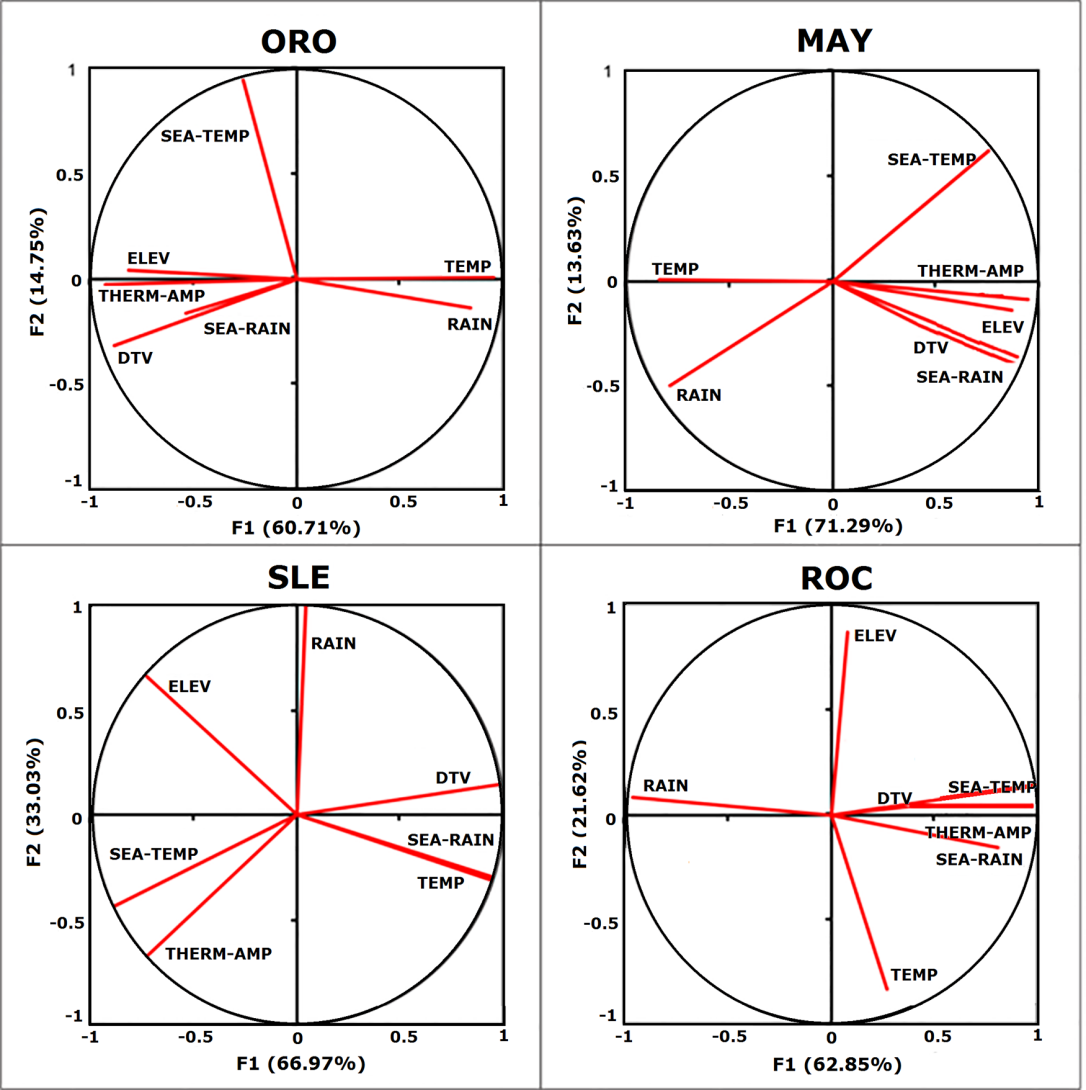
The most influential environmental variables. PCA of each disease showing which environmental variables are the most influential.

Analyzing each disease separately (Fig 2), according to PCA it was possible to perceive that the most influential factors were distinct for each arbovirus. With respect to ORO cases, the most important variables were TEMP and SEA-TEMP; for MAY: THERM-AMP and SEA-TEMP, which was similar to ORO; for SLE: RAIN and DTV, and finally for ROC, the most important variables were THERM-AMP and ELEV. Details are described in Table 2.

**Table 2:** Importance of environmental variables according to disease.

	Eigenvectors							
Environmental Variables	ORO		MAY		SLE		ROC	
	F1	F2	F1	F2	F1	F2	F1	F2
**TEMP**	**0.461**	0.006	**-0.375**	0.011	0.949	-0.316	0.129	**-0.672**
**SEA-RAIN**	-0.259	-0.156	0.369	-0.415	0.955	-0.298	0.382	-0.127
**RAIN**	0.407	-0.135	-0.352	**-0.510**	0.046	**0.999**	**-0.459**	0.068
**ELEV**	-0.394	0.040	0.387	-0.142	-0.741	0.672	0.039	**0.705**
**DTV**	-0.428	**-0.311**	0.397	-0.366	**0.990**	0.144	0.460	0.109
**THER-AMP**	**-0.448**	-0.025	**0.421**	-0.089	-0.735	**-0.678**	**0.467**	0.087
**SEA-TEMP**	-0.126	**0.926**	0.339	**0.637**	**-0.898**	-0.440	0.441	0.109

Values of PCA showing the relative influence of which environmental variables for each disease. The most influential variables (extreme values in bold) were used in the later analyzes with Maxent software.

**Fig 3:**
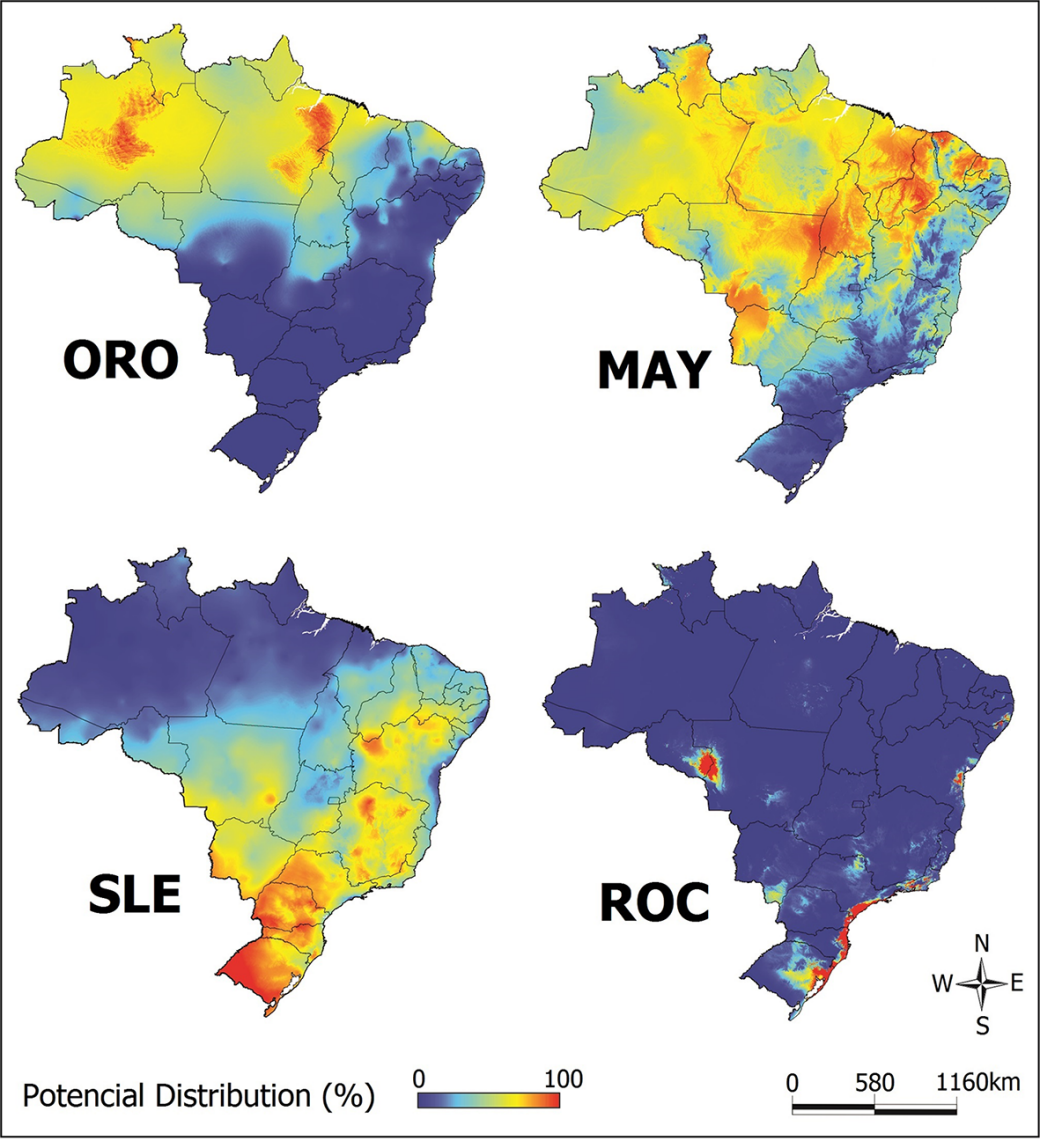
Occurrence of arboviruses. Map of the Brazilian territory showing probability of areas with ORO (AUC: 0.79), MAY (AUC: 0.76), SLE (AUC: 0.85) and ROC (AUC: 099) during outbreaks in the last five decades. The maps were built using QGis software 2.10.1.

As some variables co-varied (Fig 2), we selected only the non-covariant variables as input for analysis in Maxent software. The cut-off was four variables and was based on ROC, which presented the lowest number (four) of non-covariant variables. After selecting the four most important variables for each disease, we constructed a predictive model in Maxent (Fig 3), in order to determine what areas were most likely to present outbreaks. The contribution of each variable for each model is described in Table 3. The final model for ORO, MAY, SLE and ROC had an area under the curve of 0.79, 0.76, 0.85 and 0.99, respectively, significantly better than the random prediction (p = 0.001), indicating good performance of the model. The Maxent outputs and receiver operating characteristic curves [58] for all arboviruses are shown in S2 Fig. We observed that there is a concentration of ORO and MAY in the Northern region of Brazil, while SLE and ROC are mainly present in the South region and coastal region.

**Table 3:** Percent contribution of each variable in the final Maxent model.

Environmental Variables	Contribution (%)			
	ORO	MAY	SLE	ROC
TEMP	1.2	92.3	-	50.7
SEA-RAIN	-	-	-	-
RAIN	-	2.2	11.5	13.6
ELEV	-	-	-	25.8
DTV	3.6	-	1.4	-
THER-AMP	2.4	5.5	24.5	9.9
SEA-TEMP	92.8	0	62.7	-
**AUC index**	**0.79**	**0.76**	**0.85**	**0.99**

Percent contribution of each variable chosen for the final model obtained with Maxent. The dashes indicate those variables excluded (owing to co-variation or low influence).

**Fig 4:**
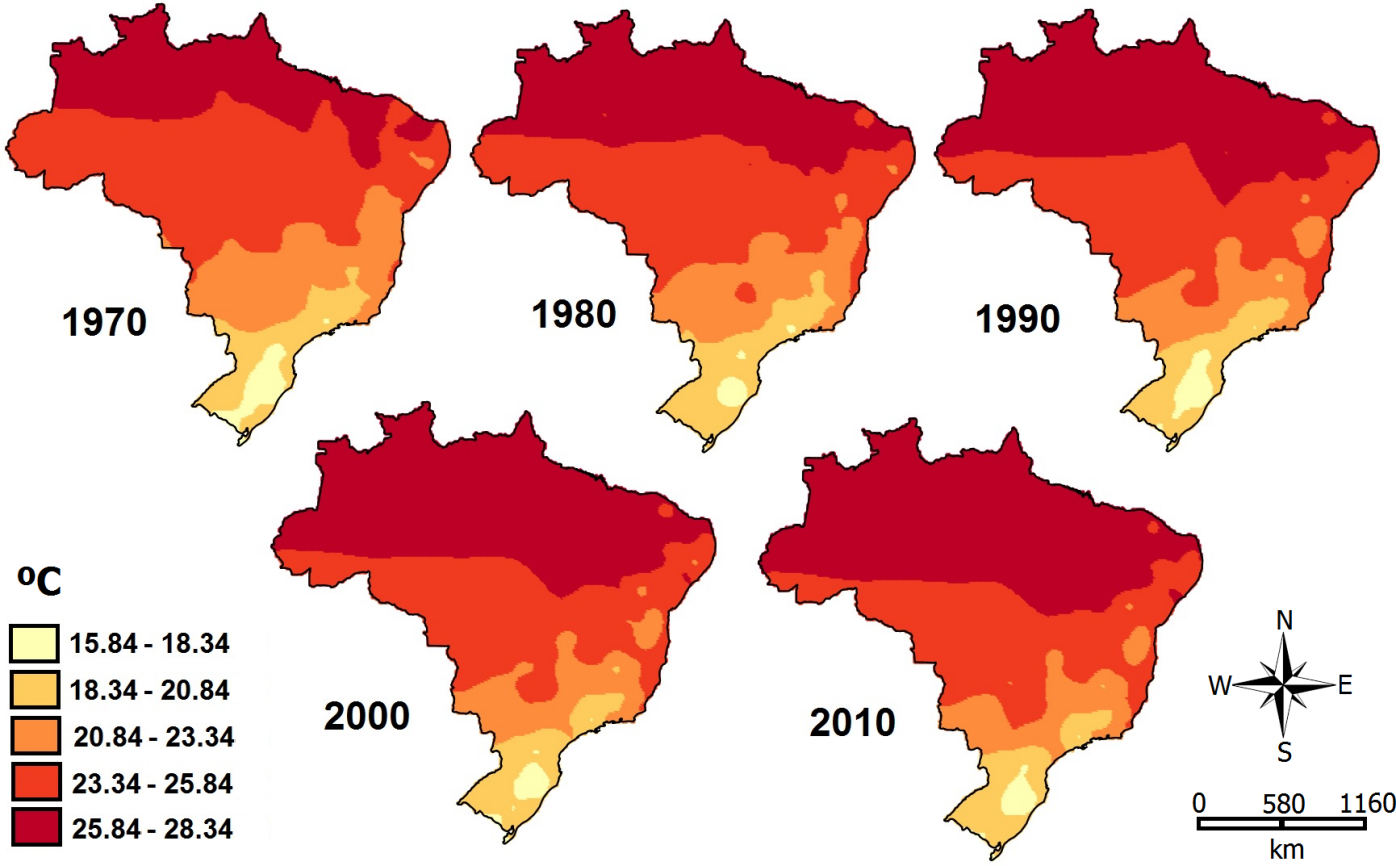
Temperature in Brazil during the last 50 years. Map showing the increase in temperature in the last five decades throughout the Brazilian territory. The maps were created using QGis software (version 2.10.1).

Most of the important variables for the distribution of all four diseases are temperature-related (TEMP, SEA-TEMP); so we analyzed the temperature situation in Brazil in the last 50 years. After analyzing the historical temperature series, and using the kriging method, we realized that there has been an increase in temperature over the decades (Fig 4), especially in the North of the country.

**Fig 5:**
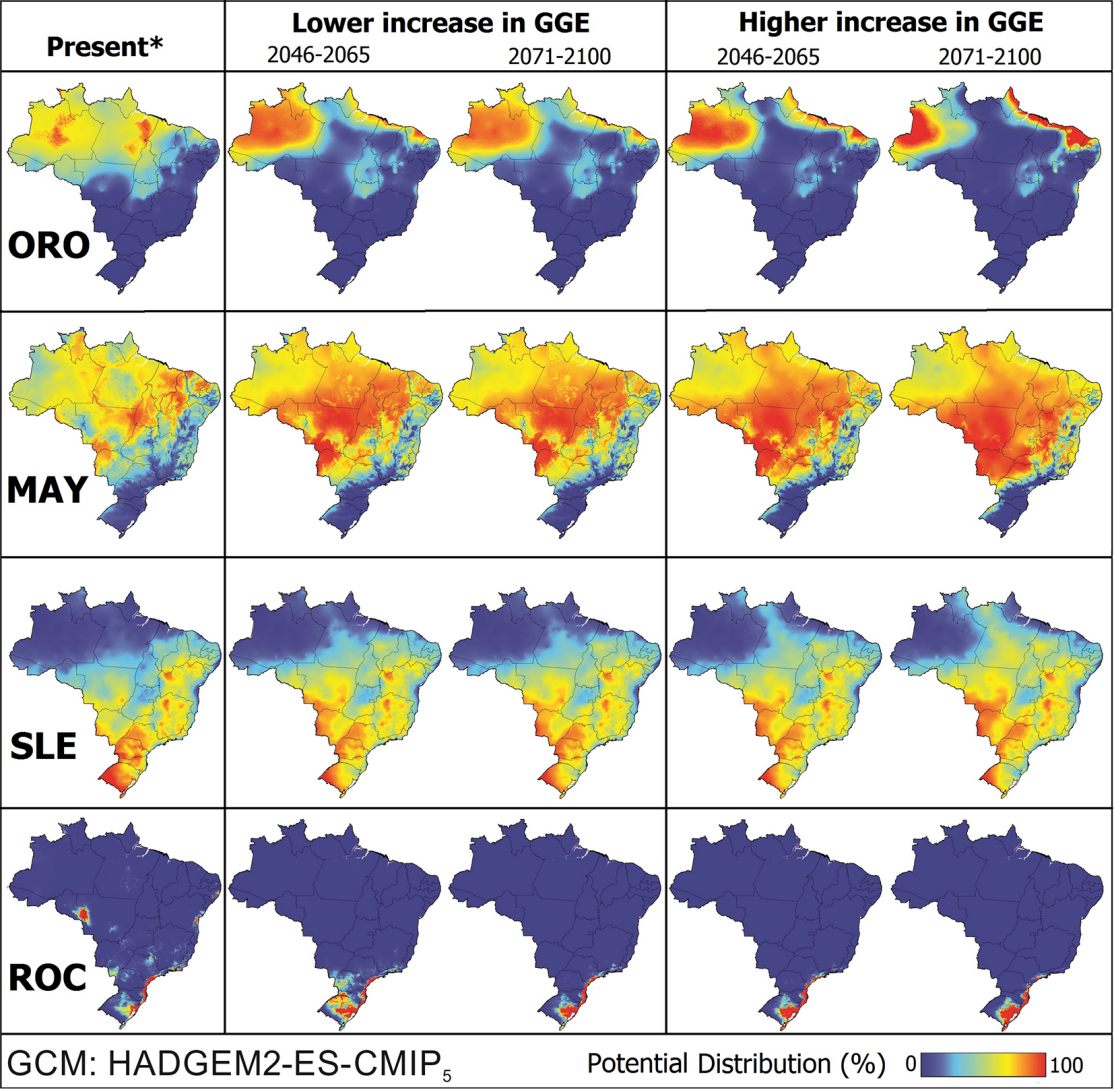
Predicted ORO, MAY, SLE and ROC range expansion in Brazil based on GCM HadGEM2-ES. The maps show the distribution under two climate change scenarios: RCP 2.6 (lower increase in greenhouse gas emissions) and RCP 8.5 (higher increase in greenhouse gas emissions). The maps were built using QGis software 2.10.1. *”Present” is the scenario in which disease outbreaks have been described, based on 1950–2010 climate data.

In the case of continuity of this scenario of temperature increase, we generated probability maps with two different climate future projections (Fig 5). The results reveal a progressively expanding areas with an increased likelihood of ORO, MAY, SLE and ROC cases, especially at the edges of the transmission areas. In scenario of high GGE it was possible to observe the increase of high risk areas for ORO and MAY, while for SLE and ROC there were no drastic changes. This fact is in agreement with our observations of temperature increase (Fig 4), which the greatest changes occurred precisely in the North region of the country, affecting mainly the distribution of ORO and MAY. We also performed these same analyzes with the GCM MIROC-5 and the results were essentially the same (S3 Fig).

## Discussion

### Modeling outbreaks in Brazil

Our analysis showed that the occurrence of outbreaks of ORO, MAY, SLE and ROC is affected differently by environmental variables, although temperature seems to be strongly associated with all of them. Theses information is quite alarming, since the temporal analyses have shown that the average temperature of some Brazilian areas has been increasing over the decades (see Fig 4). This variable presents an important constraint on the extent of expansion of these diseases throughout the country, mainly because these changes are intimately linked to vector lifecycle development and associated with the virus itself [7,59]. In addition, the IPCC report [60] points out that heat waves are more prone to occur in the next years with more frequency and duration. Furthermore, events of extreme precipitation are also most likely to be more frequent and intense on the continental surfaces, in humid tropical regions such as the North of Brazil. Consequently, climate changes may directly interfere with the distribution of the diseases evaluated in this study, particularly ORO and MAY.

The increase in temperature may also change the distribution of virus vectors, because they may migrate to other areas where conditions are favorable for proliferation. In some cases, the elevation has an inverse effect on the temperature, because generally, higher altitudes correspond with lower temperatures; nevertheless, some vectors are adaptable. In Mexico, the vector *Aedes aegypti* for example, has been found at an elevation of 2,000 meters whereas previously, it was only found in places up to 1,000 meters high [61]. For ORO and MAY outbreaks specifically, one of the factors that seems to be more related is environmental change [59]. Following deforestation in the Amazon, and subsequently, the cocoa plantation and others cultivated in the region, the vectors found an ideal spot for reproduction in the cocoa shells, and, expanded their population, spreading the virus to humans along the Amazonian roads [62]. Therefore, ORO and MAY outbreaks appear to be the result of a strong relationship between the virus and its environment, with human activity (colonization, cocoa cultivation, and subsequent environmental changes) resulting in the proliferation of the Culicidae and Ceratopogonidae mosquito families, and subsequently increasing their human contact [59,62,63].

By analyzing the results of PCA, it was observed that diseases were grouped into three main clusters: SLE, ROC and ORO+MAY (see Fig 2). The relationship between ORO and MAY occurred because both diseases are influenced by similar climatic factors, such as seasonality of temperature. Both have high-risk areas in the Northern and Central regions of the country, where the Amazonian biome predominates [5]. Moreover, both diseases are related to wild vectors, such as *Haemagogus* mosquitoes for cases of MAY and *C*. *paraensis* (Ceratopogonidae) for ORO [7]. Furthermore, although restricted to the Amazon region, these diseases have the potential to spread throughout the country and around the world, since they affect birds that can move long distances by migration or by illegal wildlife trafficking, which is very common in this region.

In relation to ROC, the factors that were most related to high-risk areas were thermal amplitude and elevation. This is in accordance with the distribution of the Atlantic Forest biome, which covers a mountainous region that shelters the wild vectors, *Aedes scapularis* and *Psorophora sp*. mosquitoes. To the best of our knowledge, no reports have explained the emergence and sudden disappearance of ROC cases in the 1970s, but it is believed that this virus is remained in a cycle where birds, including some migratory species, are the reservoirs [12]. Furthermore, in 2004, antibodies were detected among birds in southern Brazil, and the ROC virus may be circulating in different Brazilian regions, which could represent a permanent threat of disease outbreaks [7,12].

Similarly, the SLE virus can also be found in migratory birds, and it is believed to be responsible for the spread of the virus throughout the Americas, as well as other encephalitis. However, there are biological and genetic differences between isolates from North and South America [64]. Despite the rare isolates of SLE in humans in Brazil, antibodies to this virus were found in approximately 5% of the populations of the Northern and Southeastern regions [21]. The present study revealed that the most important factors for SLE were annual RAIN and DTV, which can also affect the life cycle of the *Culex* vector. For example, greater rainfall results in increasing vegetation coverage, which is the primary food source of many vertebrates that are potential hosts for the mosquitoes. However, decreased precipitation can reduce vegetation and drive both the vertebrate hosts and the mosquitoes towards human settlements, which can increase vector-human contact [2,3,65].

By analyzing the distribution maps of the diseases generated by Maxent it was observed that the probable high-risk areas were much larger than those in which the cases were detected. For example, ROC cases were reported only in the Sao Paulo state, but it is possible that there are other areas suitable for other outbreaks, such as a small region observed between the Rondonia and the Mato Grosso states (see Fig 3). This discrepancy between the reported cases and the existing cases is due mainly to the similarity of the clinical symptoms among those arboviruses, leading to an underestimation of the occurrence of ORO, MAY, SLE and ROC in Brazil. This scenario is aggravated by the lack of accurate diagnostic methods that identify which virus is acting. In cases of acute febrile illness outbreaks of MAY, SLE and ORO, for example, the laboratory procedures for diagnosing suspected cases is indispensable because these pathogens cannot be differentiated from other viral diseases, such as dengue or chikungunya, and may remain unknown [7].

As Brazil has been facing a major dengue, zika and chikungunya epidemic in recent years [66], it is quite possible that other arboviruses with similar symptoms have been underreported or confused with dengue itself. Despite the knowledge of the significant occurrence of many arboviruses in the Amazon Region, like ORO and MAY, many cases remain undiagnosed, probably because of their clinical manifestations, being usually mild and self-limited; patients generally recover completely after a few days. However, more severe cases may remain undiagnosed, especially because of the long distances to health care facilities, transport difficulties of the sample and lack of laboratories capable of conducting diagnostic tests. With regard to ORO infections, the diagnosis can easily be confused with malaria, which is endemic in that region [11,12].

According to the predictive model HadGEM2-ES, high-risk areas for all diseases may change in the next decades. For MAY, for example, practically the whole country will have adequate climatic conditions for virus transmission (see Fig 5). In some cases, such as ORO, total areas of infection will decline, but those that remain, such as the Northwestern and Northeastern regions of the country will be at increased risk. For ROC, there will be an emergent high-risk area in the South of the country, while for SLE the southern areas will become less susceptible to outbreaks. This scenario can be more or less dramatic, and depends on the levels of greenhouse gas emissions. However, this difference in emission rates will most strongly affect ORO and MAY distribution, precisely because they occupy the Northern areas of Brazil and are more susceptible to temperature changes (see Fig 4). Climatic change also affect human activity and the migration of the people, as well as the redistribution of the vectors, and a more favorable environment for the propagation of arboviruses [59]. Therefore, future risk estimations should consider those factors. Nevertheless, despite all efforts and cumulated knowledge in the literature, it is still remains difficult to identify the main cause of an outbreak [67] and to determine the most efficient technique(s) for protecting humans from these viruses. However, our findings indicate that temperature-related variables appear to play a central role in the epidemiology of culicid-vectored arboviruses.

### Limitations

This study has limitations, since the projections presented here are processed on the assumption that the other variables remained stable. The human population size, for example, was considered as constant in our models. Moreover, we did not consider host migration (of both birds and humans), and the deforestation rate, which are important factors in determining the outbreaks of ORO and SLE. Yet, other factors that were not part of our model could change over the given time period: For example, the quality of vector surveillance, clinical case detection, and the development of some vaccine. Besides that, we did not take into account the distribution areas of the vectors, which are essential for the transmission, but very difficult to estimate. Another point is we used linear extrapolation to predict the distributions of each disease, and our model did not account for non-linear phenomena. For example, the models would fail if temperature increased to the point that it changed the vector transmission rates.

Another limitation is the supposed underreporting of cases and the confusion with other diseases. These neglected arboviruses may be circulating in asymptomatic patients or misdiagnosed and it is not possible to know their exact distribution; this affects directly the predictive power of our model. The model itself also has an intrinsic limitation, because it assumes that the increase in temperature can only be owing to GGE. We know that this is not the only possibility in nature, so this is a simplified premise that limitates interpretations. Although there are a number of constraints to our model, it is an important first step in trying to predict the emergence of these neglected arboviruses. It also serves to warn the vigilant surveillance health that arboviruses may remain longly underestimated before outbreak arises.

### Conclusions

In summary, environmental factors can directly affect the distribution of ORO, MAY, SLE and ROC. Among them, temperature is a central variable that determines the distribution of high-risk areas. As the average temperature of some Brazilian areas has augmented significantly over the last 50 years, a better understanding of the biology of neglected arboviruses, their interactions and consequences in the ecosystem and climatic factors is needed. The four diseases addressed in the present work are clearly a latent menace to the public health and thus should be promptly included in the health programs agenda. Accurate detection and diagnosing are fundamental steps for developing efficient control measures. These four apparently similar arboviruses are differently affected by environmental factors, and these differences are probably linked to the vector’s lifecycle or associated with the virus itself. We can also conclude that our mathematical and statistical approach allowed us to further describe peculiar environmental elements in the epidemiology of these neglected diseases. Even though, the approach is limited and we suggest that next studies should be multidisciplinary and comprehensive so that they include vector distribution, components of natural cycle and actual disease incidence.

Overall, this work provides useful indications about the dynamics of those arboviruses across the country. Our results suggest that high-risk areas may change in the coming years, being more pronounced with high GGE rates in the northern region of the country. This is the first spatio-temporal study of these arboviral diseases. However, there are gaps in available knowledge to scientifically predict future occurrences of large epidemics. Due to the epidemiological and entomological situation of several continents, there is evidence of aggravation of this current scenario, because there is great difficulty in eliminating or controlling the risk factors of the diseases.

## Supporting information

S1 Fig Raw temperatures. Maps of raw temperature projections from the GCMs models.
(TIF)

S2 Fig Maxent outputs. Detailed output data and receiver operating characteristic (ROC) curves for each disease.
(PDF)

S3 Fig Predicted ORO, MAY, SLE and ROC range expansion in Brazil based on GCM MIROC-5. The maps show the distribution under two climate change scenarios: RCP 2.6 (lower increase in greenhouse gas emissions) and RCP 8.5 (higher increase in greenhouse gas emissions). The maps were built using QGis software 2.10.1. *”Present” is the scenario in which disease outbreaks have been described, based on 1950–2010 climate data.
(TIF)
