# Supplementary figures and images for: Impact of environmental factors on neglected emerging arboviral diseases
Source: PLoS Negl Trop Dis. 2017 Sep 27;11(9):e0005959. doi: 10.1371/journal.pntd.0005959 (PMC5633201; doi:10.1371/journal.pntd.0005959)

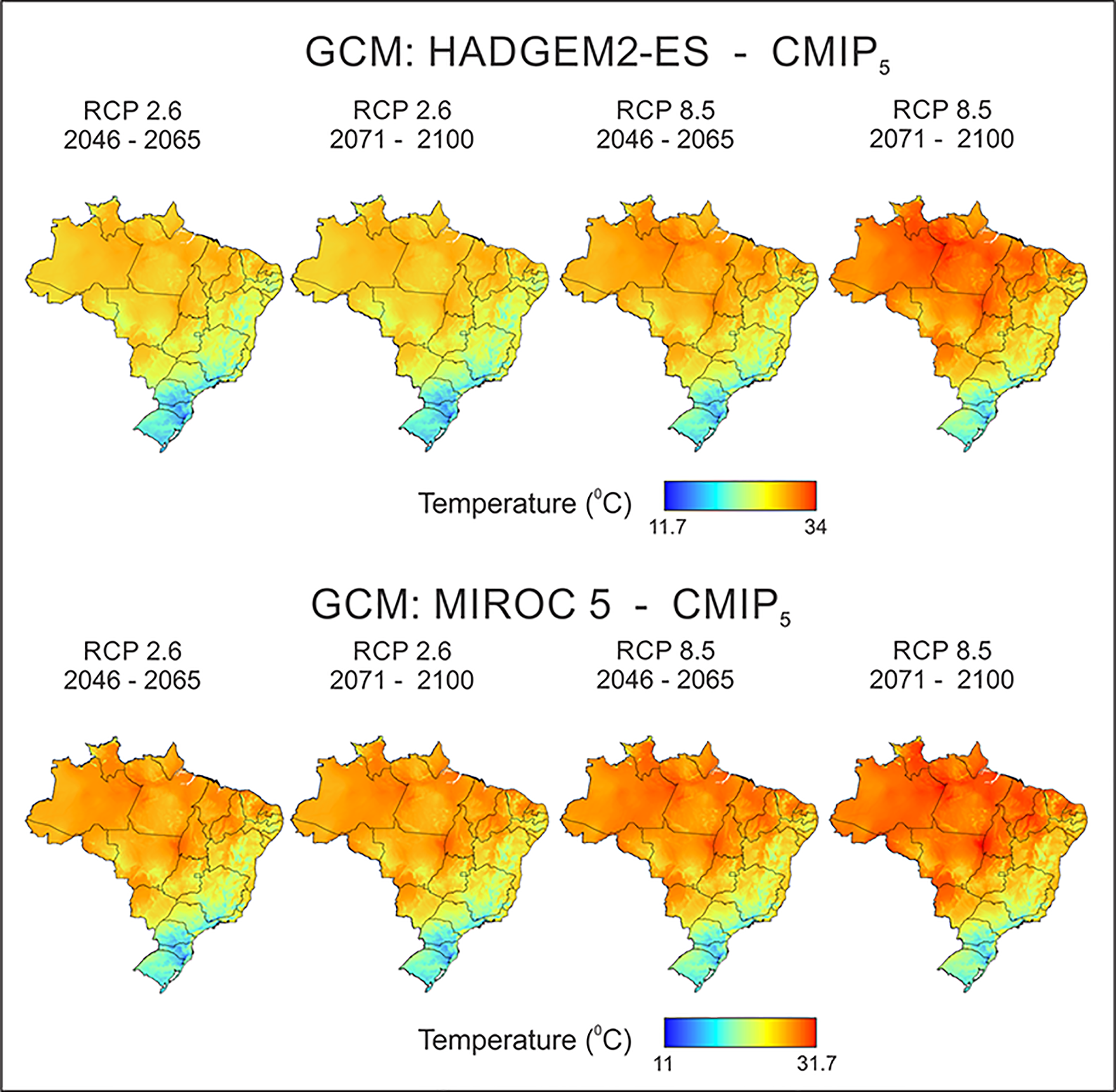

Supplement: S1 Fig — Maps of raw temperature projections from the GCMs models. (TIF) [file pntd.0005959.s001.tif]

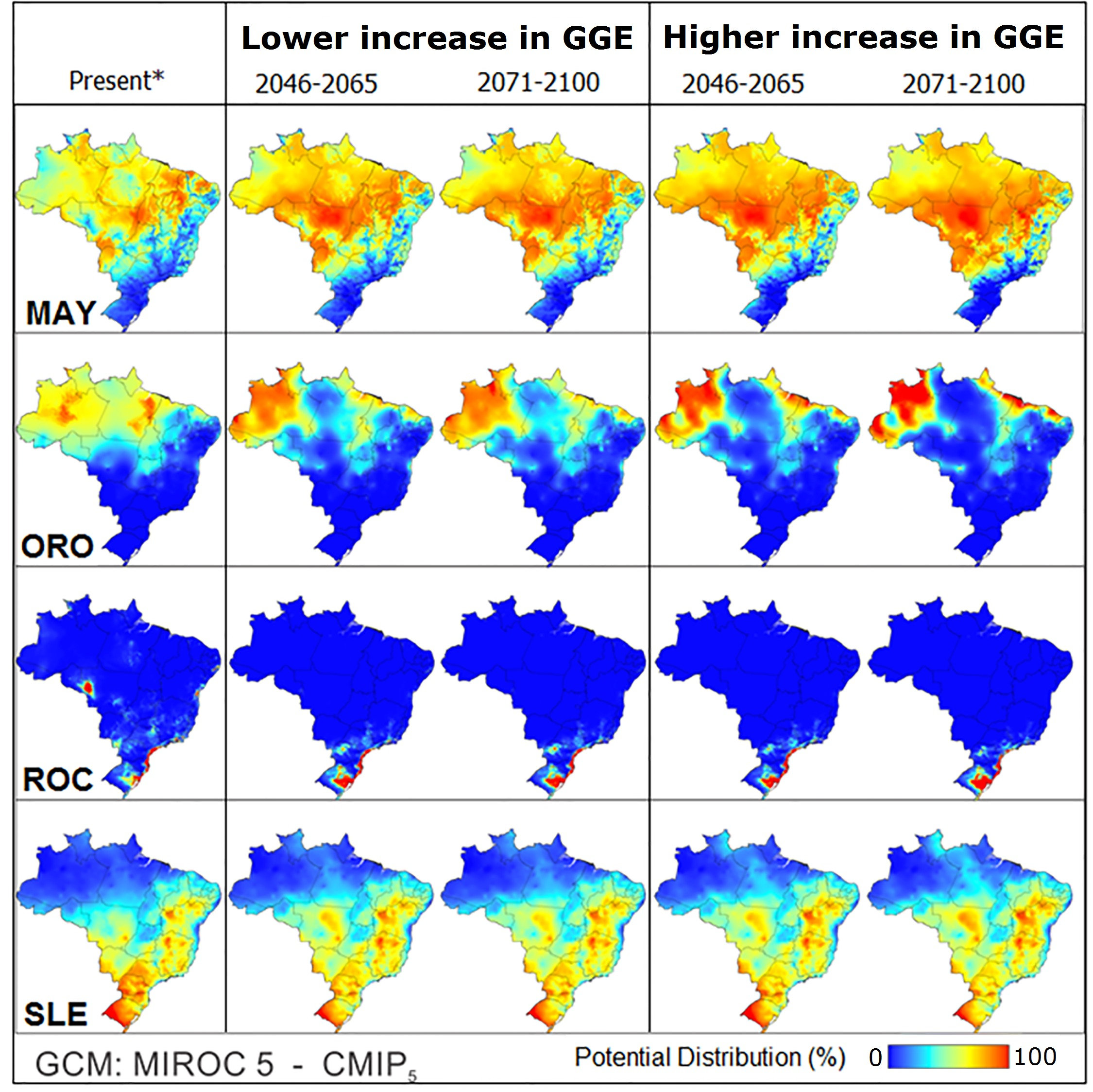

Supplement: S3 Fig — The maps show the distribution under two climate change scenarios: RCP 2.6 (lower increase in greenhouse gas emissions) and RCP 8.5 (higher increase in greenhouse gas emissions). The maps were built using QGis software 2.10.1. *”Present” is the scenario in which disease outbreaks have been described, based on 1950–2010 climate data. (TIF) [file pntd.0005959.s003.tif]
