# Supplementary material for: Impact of environmental factors on neglected emerging arboviral diseases
Source: PLoS Negl Trop Dis. 2017 Sep 27;11(9):e0005959. doi: 10.1371/journal.pntd.0005959 (PMC5633201; doi:10.1371/journal.pntd.0005959)
Supplement: S2 Fig — Detailed output data and receiver operating characteristic (ROC) curves for each disease. (PDF) [file pntd.0005959.s002.pdf]

Maxent model for Oropouche

This page contains some analysis of the Maxent model for Oropouche, created Fri Nov 11 01:04:51 BRST 2016 using Maxent version 3.3.3k. If you would like to do further analyses, the raw data used here is linked to at the end of this page.

Analysis of omission/commission

The following picture shows the omission rate and predicted area as a function of the cumulative threshold. The omission rate is is calculated both on the training presence records, and (if test data are used) on the test records. The omission rate should be close to the predicted omission, because of the definition of the cumulative threshold.

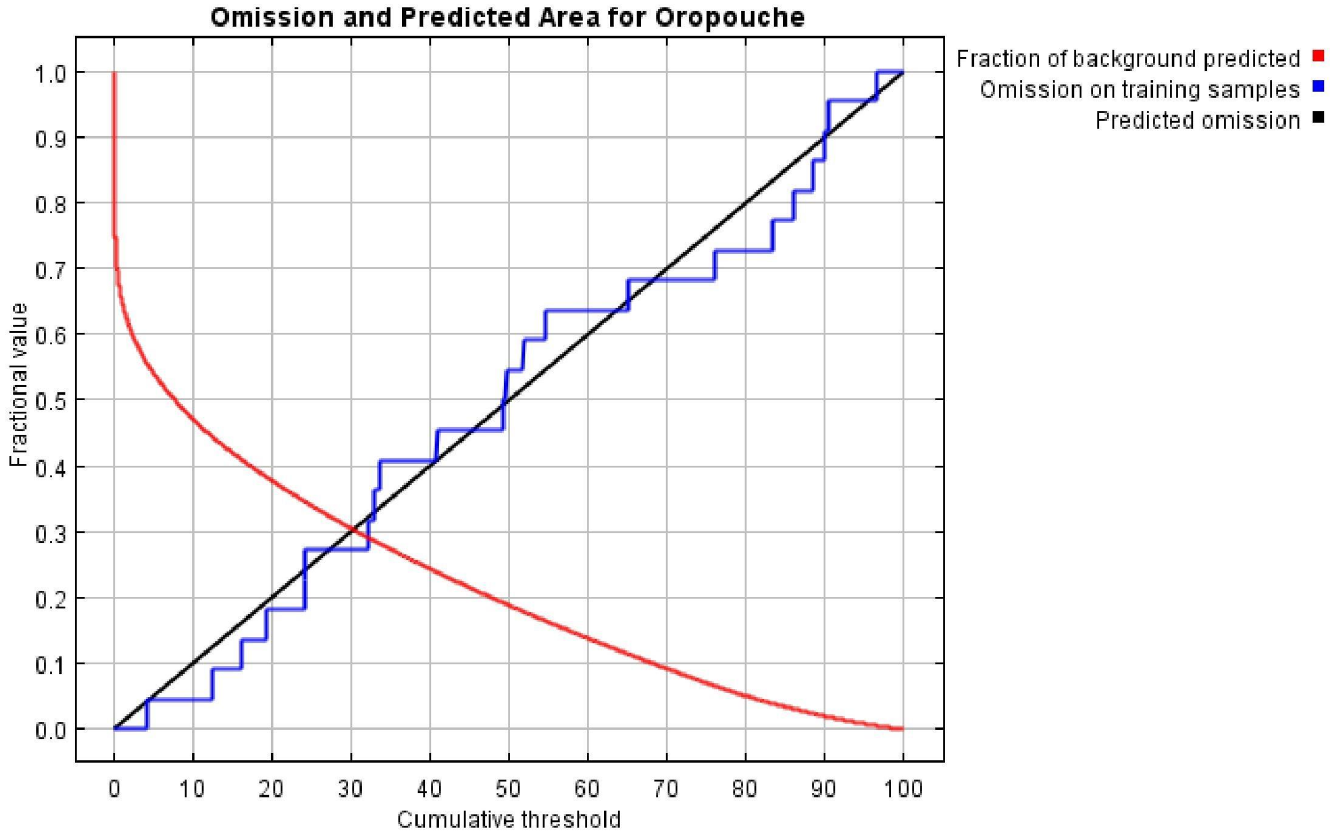

The next picture is the receiver operating characteristic (ROC) curve for the same data. Note that the specificity is defined using predicted area, rather than true commission (see the paper by Phillips, Anderson and Schapire cited on the help page for discussion of what this means). This implies that the maximum achievable AUC is less than 1. If test data is drawn from the Maxent distribution itself, then the maximum possible test AUC would be 0.780 rather than 1; in practice the test AUC may exceed this bound.

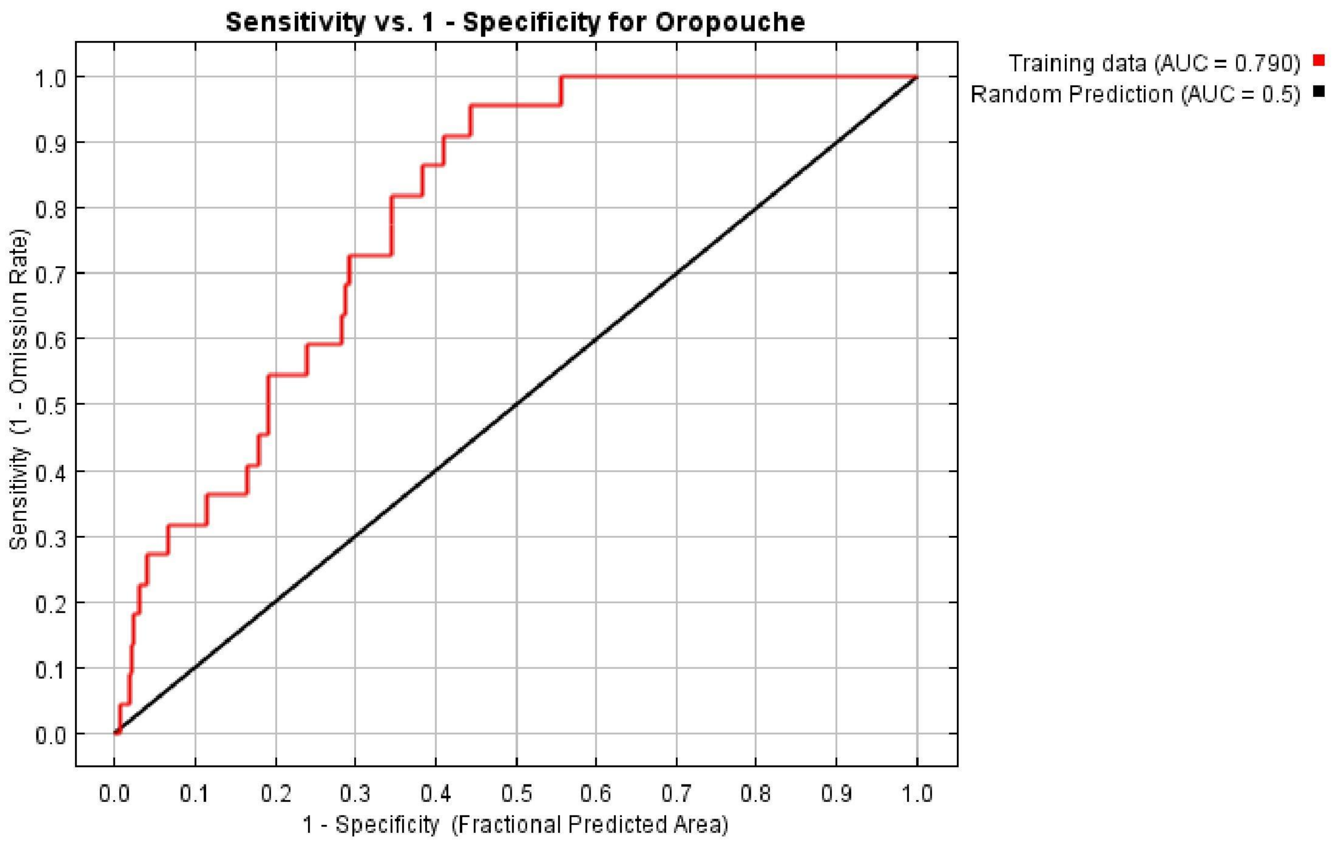

Some common thresholds and corresponding omission rates are as follows. If test data are available, binomial probabilities are calculated exactly if the number of test samples is at most 25, otherwise using a normal approximation to the binomial. These are 1-sided p-values for the null hypothesis that test points are predicted no better than by a random prediction with the same fractional predicted area. The "Balance" threshold minimizes 6 * training omission rate + .04 * cumulative threshold + 1.6 * fractional predicted area.

Cumulative threshold	Logistic threshold	Description	Fractional predicted area	Training omission rate
1.000	0.093	Fixed cumulative value 1	0.649	0.000
5.000	0.246	Fixed cumulative value 5	0.541	0.045
10.000	0.321	Fixed cumulative value 10	0.471	0.045
4.157	0.229	Minimum training presence	0.556	0.000
16.145	0.391	10 percentile training presence	0.410	0.091
32.119	0.466	Equal training sensitivity and specificity	0.291	0.273
12.383	0.355	Maximum training sensitivity plus specificity	0.445	0.045
2.837	0.182	Balance training omission, predicted area and threshold value	0.584	0.000
4.067	0.227	Equate entropy of thresholded and original distributions	0.557	0.000

Pictures of the model

This is a representation of the Maxent model for Oropouche. Warmer colors show areas with better predicted conditions. White dots show the presence locations used for training, while violet dots show test locations. Click on the image for a full-size version.

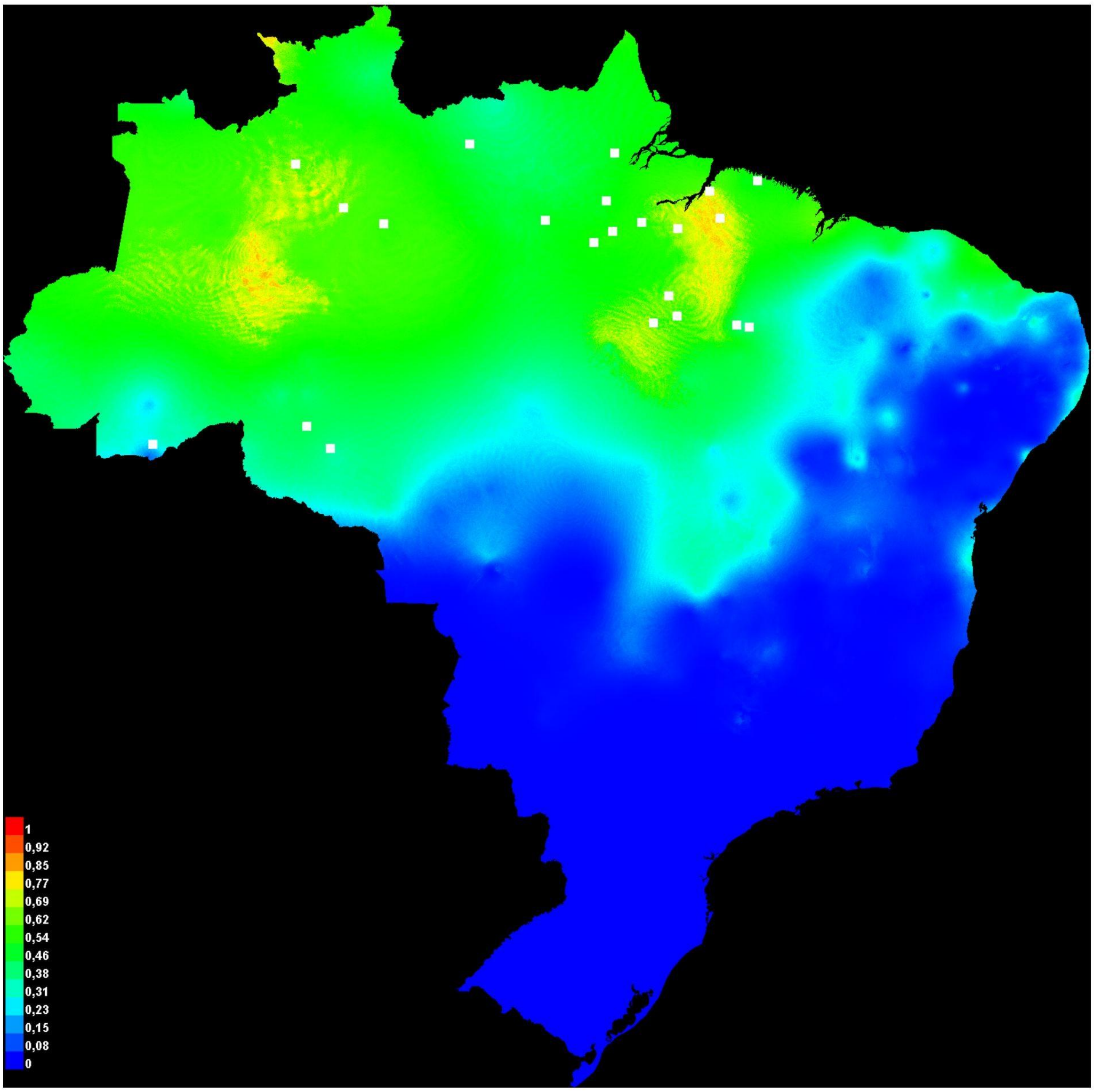

Click here to interactively explore this prediction using the Explain tool. If clicking from your browser does not succeed in starting the tool, try running the script in C:\Users\THIAGO AZEVEDO\Desktop\Elcefalites\Modelos\Modelos_atual_discriminado\Oropouche_explain.bat directly. This tool requires the environmental grids to be small enough that they all fit in memory.

Response curves

These curves show how each environmental variable affects the Maxent prediction. The curves show how the logistic prediction changes as each environmental variable is varied, keeping all other environmental variables at their average sample value. Click on a response curve to see a larger version. Note that the curves can be hard to interpret if you have strongly correlated variables, as the model may depend on the

correlations in ways that are not evident in the curves. In other words, the curves show the marginal effect of changing exactly one variable, whereas the model may take advantage of sets of variables changing together.

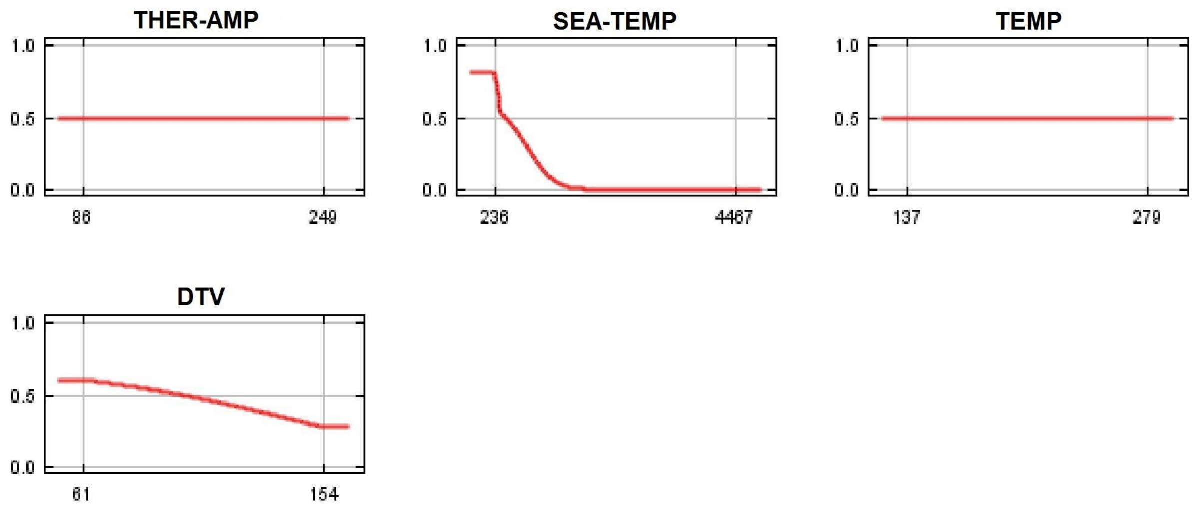

In contrast to the above marginal response curves, each of the following curves represents a different model, namely, a Maxent model created using only the corresponding variable. These plots reflect the dependence of predicted suitability both on the selected variable and on dependencies induced by correlations between the selected variable and other variables. They may be easier to interpret if there are strong correlations between variables.

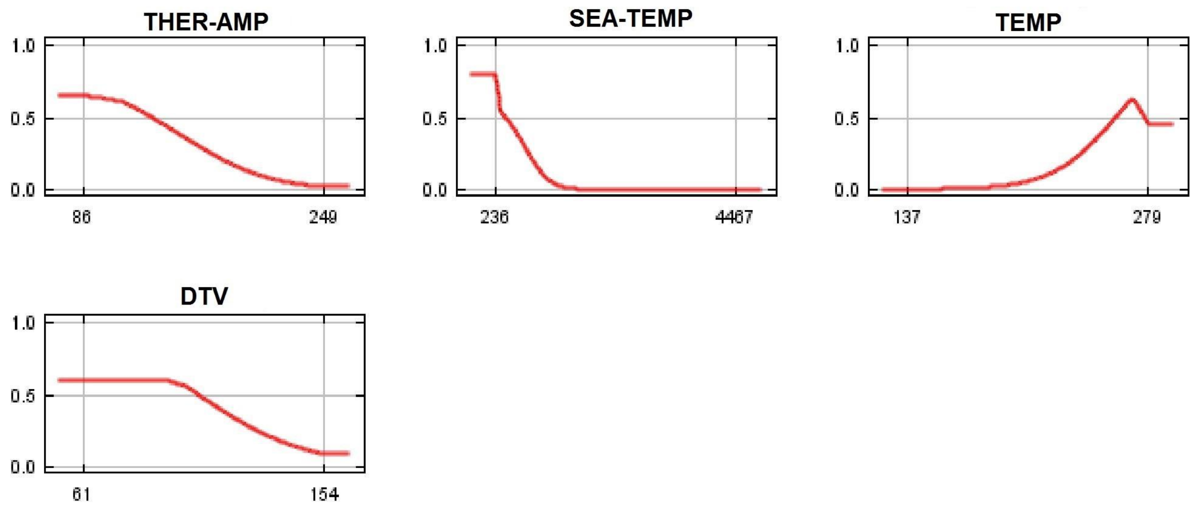

Analysis of variable contributions

The following table gives estimates of relative contributions of the environmental variables to the Maxent model. To determine the first estimate, in each iteration of the training algorithm, the increase in regularized gain is added to the contribution of the corresponding variable, or subtracted from it if the change to the absolute value of lambda is negative. For the second estimate, for each environmental variable in turn, the values of that variable on training presence and background data are randomly permuted. The model is reevaluated on the permuted data, and the resulting drop in training AUC is shown in the table, normalized to percentages. As with the variable jackknife, variable contributions should be interpreted with caution when the predictor variables are correlated.

Variable	Percent contribution	Permutation importance
SEA-TEMP	92.8	89.1
DTV	3.6	10.9
THER-AMP	2.4	0.1
TEMP	1.2	0

Raw data outputs and control parameters

The data used in the above analysis is contained in the next links. Please see the Help button for more information on these.
The model applied to the training environmental layers
The coefficients of the model
The omission and predicted area for varying cumulative and raw thresholds
The prediction strength at the training and (optionally) test presence sites
Results for all species modeled in the same Maxent run, with summary statistics and (optionally) jackknife results

Regularized training gain is 0.584, training AUC is 0.790, unregularized training gain is 0.665.
Algorithm converged after 140 iterations (1 seconds).

The follow settings were used during the run:
22 presence records used for training.
10022 points used to determine the Maxent distribution (background points and presence points).
Environmental layers used (all continuous): amplitermica sazonalidadetemp tempanual vardiurntemp
Regularization values: linear/quadratic/product: 0.404, categorical: 0.250, threshold: 1.780, hinge: 0.500
Feature types used: linear quadratic hinge
responsecurves: true
outputdirectory: C:\Users\THIAGO AZEVEDO\Desktop\Elcefalites\Modelos\Modelos_atual_discriminado
samplesfile: C:\Users\THIAGO AZEVEDO\Desktop\Elcefalites\Modelos\Surtos\Oropouche.csv
environmentallayers: C:\Users\THIAGO AZEVEDO\Desktop\Elcefalites\Raster\dados
Command line used:

Command line to repeat this species model: java density.MaxEnt nowarnings noprefixes -E "" -E Oropouche responsecurves "outputdirectory=C:\Users\THIAGO AZEVEDO\Desktop\Elcefalites\Modelos\Modelos_atual_discriminado" "samplesfile=C:\Users\THIAGO AZEVEDO\Desktop\Elcefalites\Modelos\Surtos\Oropouche.csv" "environmentallayers=C:\Users\THIAGO AZEVEDO\Desktop\Elcefalites\Raster\dados" -N altitude -N precipanual

Maxent model for Mayaro

This page contains some analysis of the Maxent model for Mayaro, created Fri Nov 11 00:59:22 BRST 2016 using Maxent version 3.3.3k. If you would like to do further analyses, the raw data used here is linked to at the end of this page.

Analysis of omission/commission

The following picture shows the omission rate and predicted area as a function of the cumulative threshold. The omission rate is is calculated both on the training presence records, and (if test data are used) on the test records. The omission rate should be close to the predicted omission, because of the definition of the cumulative threshold.

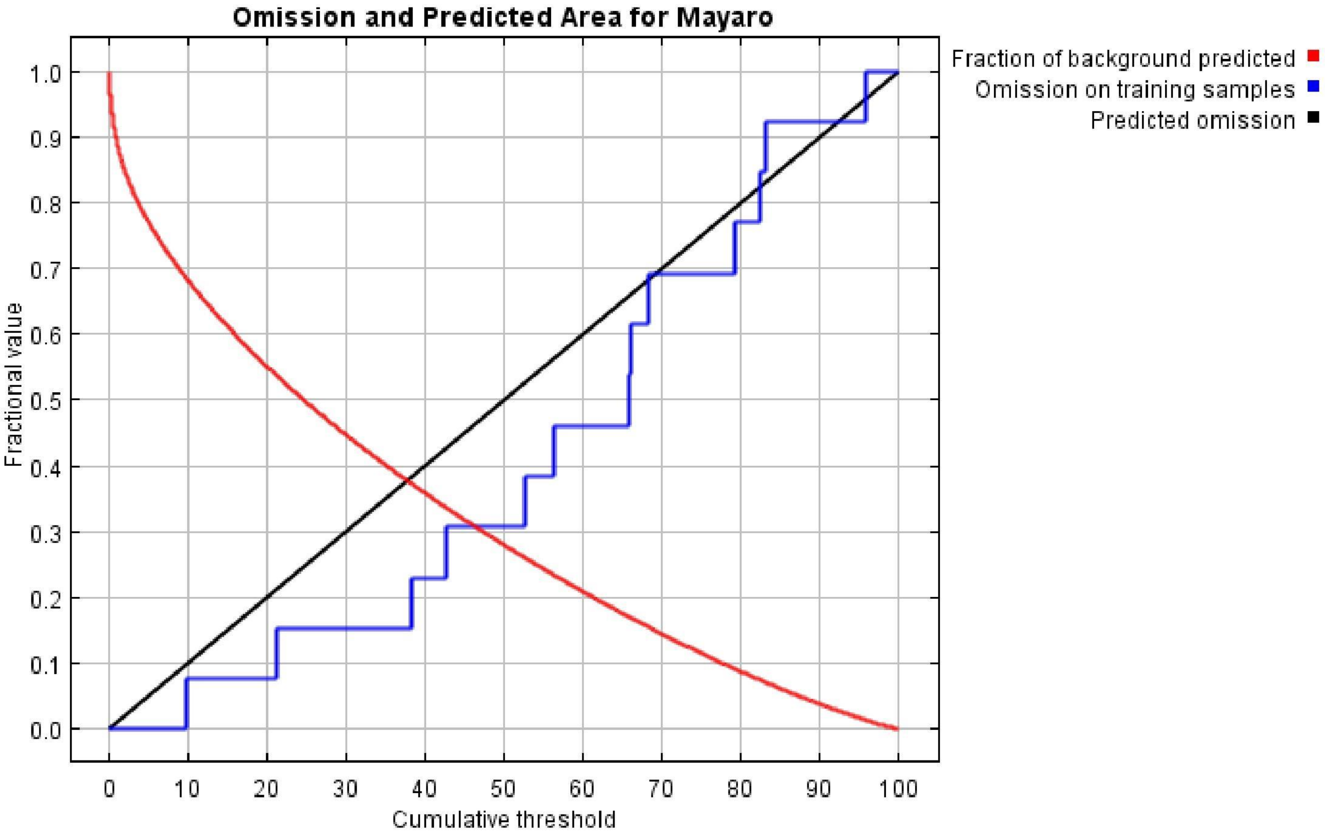

The next picture is the receiver operating characteristic (ROC) curve for the same data. Note that the specificity is defined using predicted area, rather than true commission (see the paper by Phillips, Anderson and Schapire cited on the help page for discussion of what this means). This implies that the maximum achievable AUC is less than 1. If test data is drawn from the Maxent distribution itself, then the maximum possible test AUC would be 0.677 rather than 1; in practice the test AUC may exceed this bound.

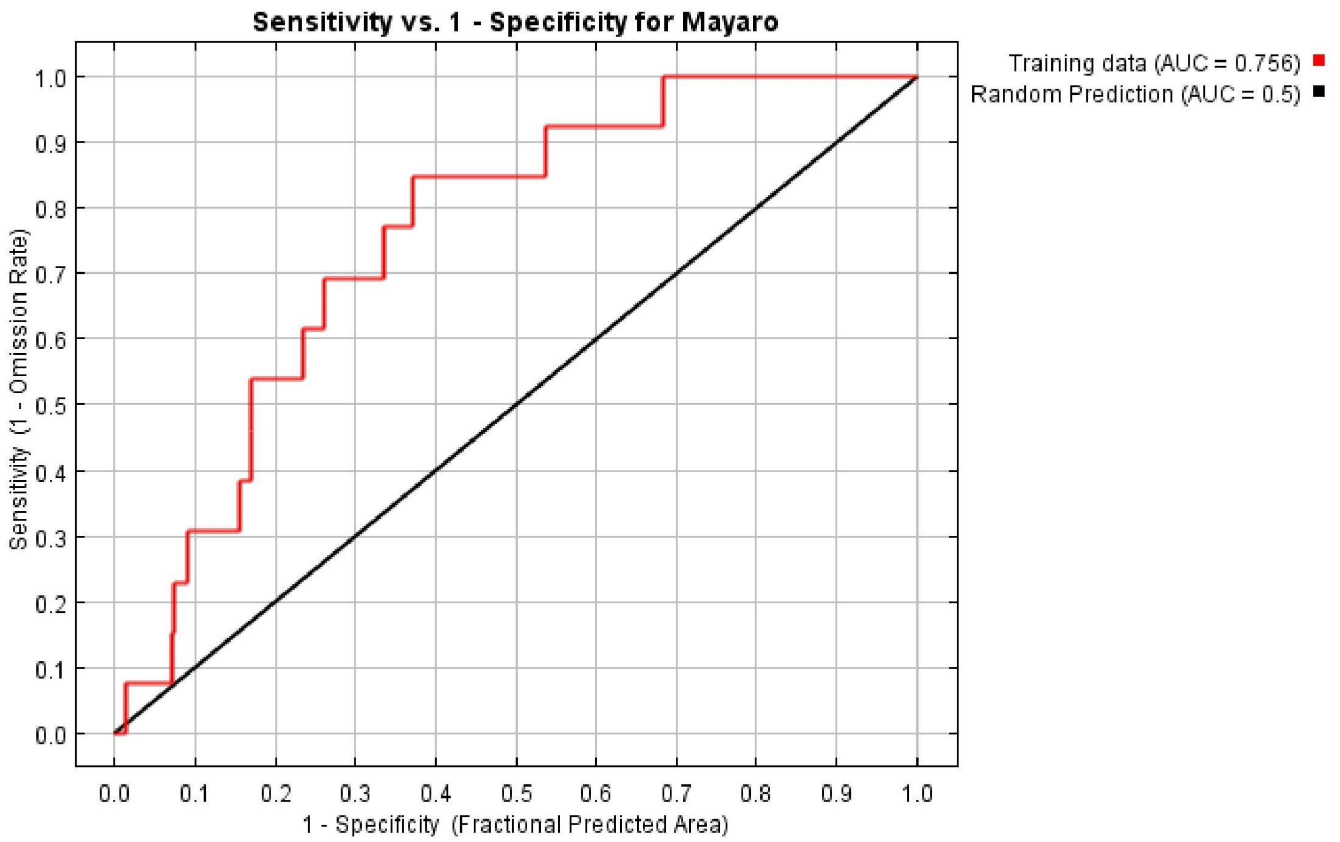

Some common thresholds and corresponding omission rates are as follows. If test data are available, binomial probabilities are calculated exactly if the number of test samples is at most 25, otherwise using a normal approximation to the binomial. These are 1-sided p-values for the null hypothesis that test points are predicted no better than by a random prediction with the same fractional predicted area. The "Balance" threshold minimizes 6 * training omission rate + .04 * cumulative threshold + 1.6 * fractional predicted area.

Cumulative threshold	Logistic threshold	Description	Fractional predicted area	Training omission rate
1.000	0.128	Fixed cumulative value 1	0.890	0.000
5.000	0.279	Fixed cumulative value 5	0.770	0.000
10.000	0.344	Fixed cumulative value 10	0.682	0.077
9.809	0.342	Minimum training presence	0.685	0.000
21.129	0.419	10 percentile training presence	0.538	0.077
46.389	0.511	Equal training sensitivity and specificity	0.308	0.308
38.377	0.488	Maximum training sensitivity plus specificity	0.372	0.154
3.520	0.244	Balance training omission, predicted area and threshold value	0.803	0.000
3.397	0.240	Equate entropy of thresholded and original distributions	0.806	0.000

Pictures of the model

This is a representation of the Maxent model for Mayaro. Warmer colors show areas with better predicted conditions. White dots show the presence locations used for training, while violet dots show test locations. Click on the image for a full-size version.

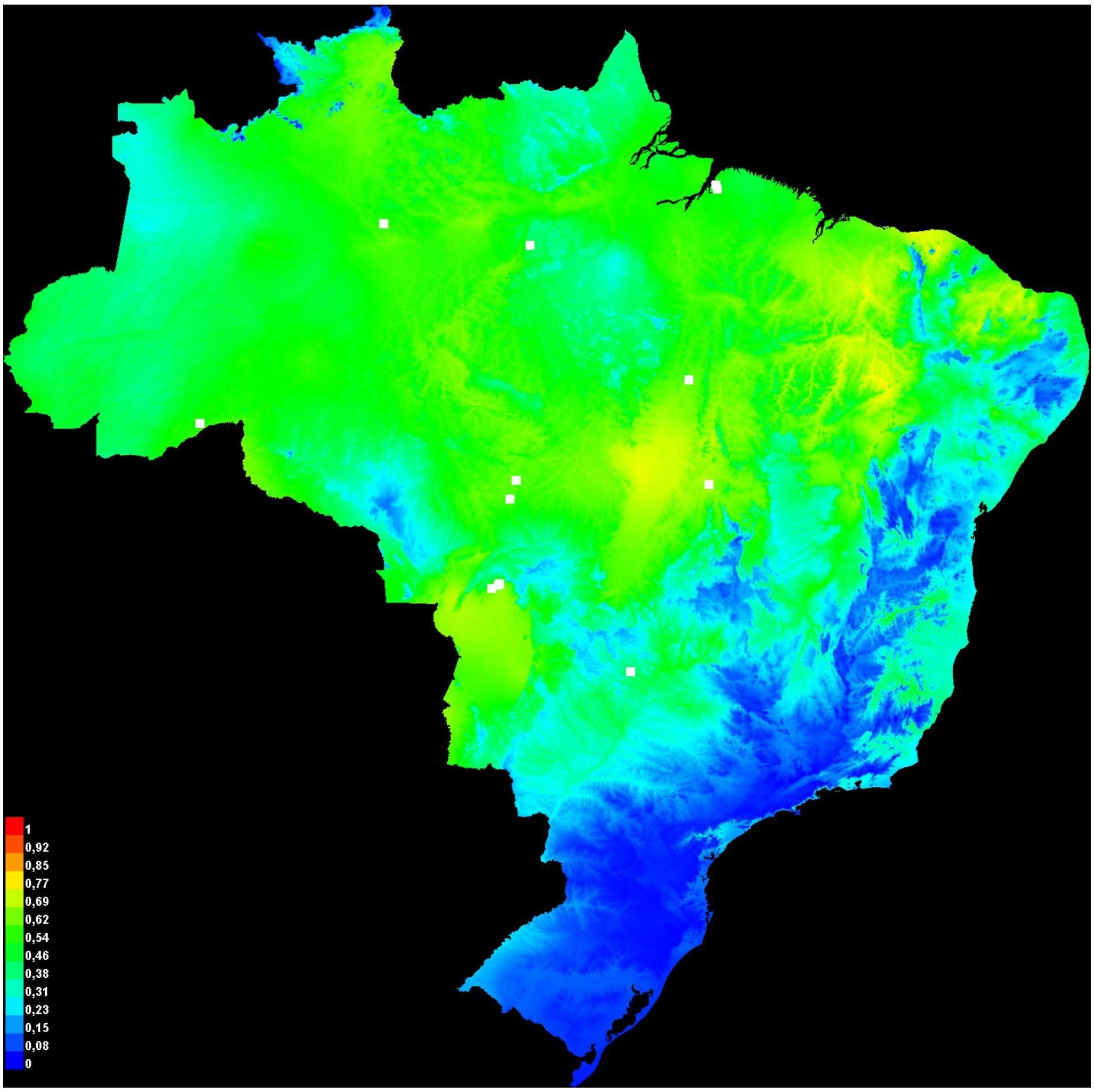

Click here to interactively explore this prediction using the Explain tool. If clicking from your browser does not succeed in starting the tool, try running the script in C:\Users\THIAGO AZEVEDO\Desktop\Elcefalites\Modelos\Modelos_atual_discriminado\Mayaro_explain.bat directly. This tool requires the environmental grids to be small enough that they all fit in memory.

Response curves

These curves show how each environmental variable affects the Maxent prediction. The curves show how the logistic prediction changes as each environmental variable is varied, keeping all other environmental variables at their average sample value. Click on a response curve to see a larger version. Note that the curves can be hard to interpret if you have strongly correlated variables, as the model may depend on the

correlations in ways that are not evident in the curves. In other words, the curves show the marginal effect of changing exactly one variable, whereas the model may take advantage of sets of variables changing together.

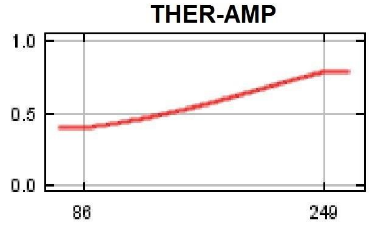

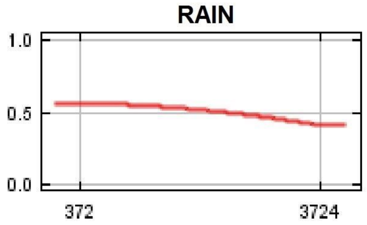

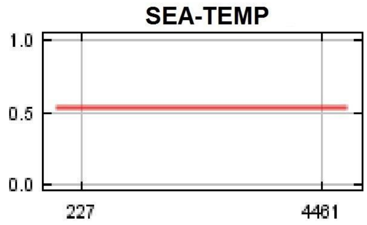

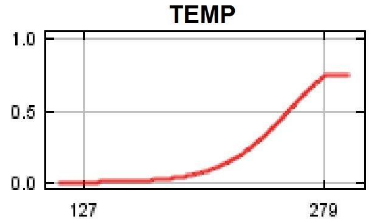

In contrast to the above marginal response curves, each of the following curves represents a different model, namely, a Maxent model created using only the corresponding variable. These plots reflect the dependence of predicted suitability both on the selected variable and on dependencies induced by correlations between the selected variable and other variables. They may be easier to interpret if there are strong correlations between variables.

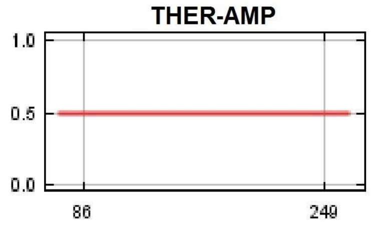

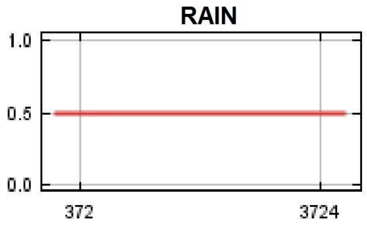

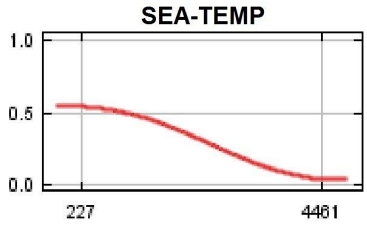

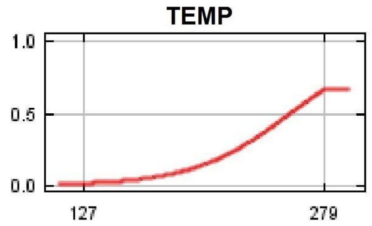

Analysis of variable contributions

The following table gives estimates of relative contributions of the environmental variables to the Maxent model. To determine the first estimate, in each iteration of the training algorithm, the increase in regularized gain is added to the contribution of the corresponding variable, or subtracted from it if the change to the absolute value of lambda is negative. For the second estimate, for each environmental variable in turn, the values of that variable on training presence and background data are randomly permuted. The model is reevaluated on the permuted data, and the resulting drop in training AUC is shown in the table, normalized to percentages. As with the variable jackknife, variable contributions should be interpreted with caution when the predictor variables are correlated.

Variable	Percent contribution	Permutation importance
TEMP	92.3	60.1
THER-AMP	5.5	30.5
RAIN	2.2	9.4
SEA-TEMP	0	0

Raw data outputs and control parameters

The data used in the above analysis is contained in the next links. Please see the Help button for more information on these.
The model applied to the training environmental layers
The coefficients of the model
The omission and predicted area for varying cumulative and raw thresholds
The prediction strength at the training and (optionally) test presence sites
Results for all species modeled in the same Maxent run, with summary statistics and (optionally) jackknife results

Regularized training gain is 0.215, training AUC is 0.756, unregularized training gain is 0.370.
Algorithm converged after 120 iterations (0 seconds).

The follow settings were used during the run:
13 presence records used for training.
10013 points used to determine the Maxent distribution (background points and presence points).
Environmental layers used (all continuous): amplitermica precipanual sazonalidadetemp tempanual
Regularization values: linear/quadratic/product: 0.671, categorical: 0.393, threshold: 1.870, hinge: 0.500
Feature types used: linear quadratic
responsecurves: true
outputdirectory: C:\Users\THIAGO AZEVEDO\Desktop\Elcefalites\Modelos\Modelos_atual_discriminado
samplesfile: C:\Users\THIAGO AZEVEDO\Desktop\Elcefalites\Modelos\Surtos\Mayaro.csv
environmentallayers: C:\Users\THIAGO AZEVEDO\Desktop\Elcefalites\Raster\dados
Command line used:

Command line to repeat this species model: java density.MaxEnt nowarnings noprefixes -E "" -E Mayaro responsecurves "outputdirectory=C:\Users\THIAGO AZEVEDO\Desktop\Elcefalites\Modelos\Modelos_atual_discriminado" "samplesfile=C:\Users\THIAGO AZEVEDO\Desktop\Elcefalites\Modelos\Surtos\Mayaro.csv" "environmentallayers=C:\Users\THIAGO AZEVEDO\Desktop\Elcefalites\Raster\dados" -N altitude -N vardiurntemp

Maxent model for Saint_Louis

This page contains some analysis of the Maxent model for Saint_Louis, created Fri Nov 11 01:15:24 BRST 2016 using Maxent version 3.3.3k. If you would like to do further analyses, the raw data used here is linked to at the end of this page.

Analysis of omission/commission

The following picture shows the omission rate and predicted area as a function of the cumulative threshold. The omission rate is is calculated both on the training presence records, and (if test data are used) on the test records. The omission rate should be close to the predicted omission, because of the definition of the cumulative threshold.

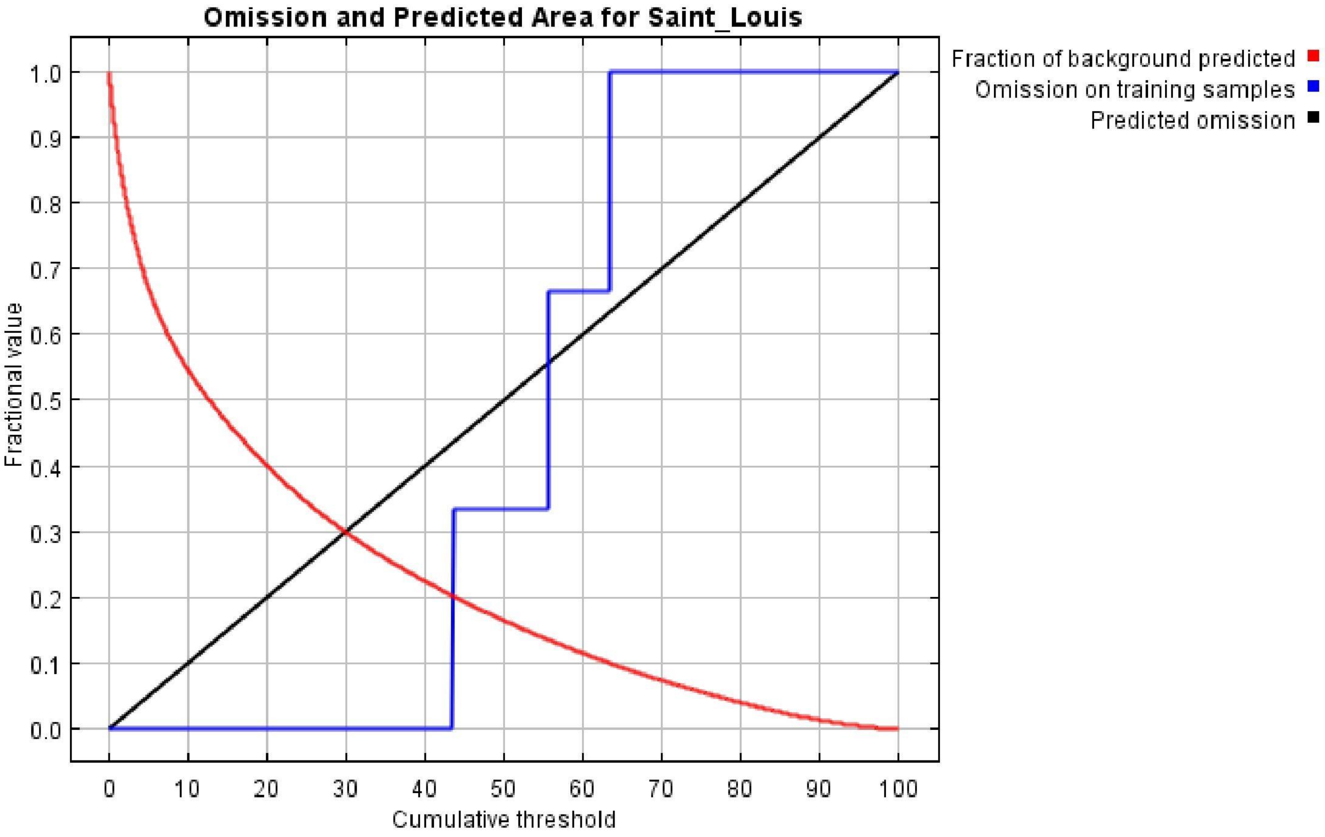

The next picture is the receiver operating characteristic (ROC) curve for the same data. Note that the specificity is defined using predicted area, rather than true commission (see the paper by Phillips, Anderson and Schapire cited on the help page for discussion of what this means). This implies that the maximum achievable AUC is less than 1. If test data is drawn from the Maxent distribution itself, then the maximum possible test AUC would be 0.771 rather than 1; in practice the test AUC may exceed this bound.

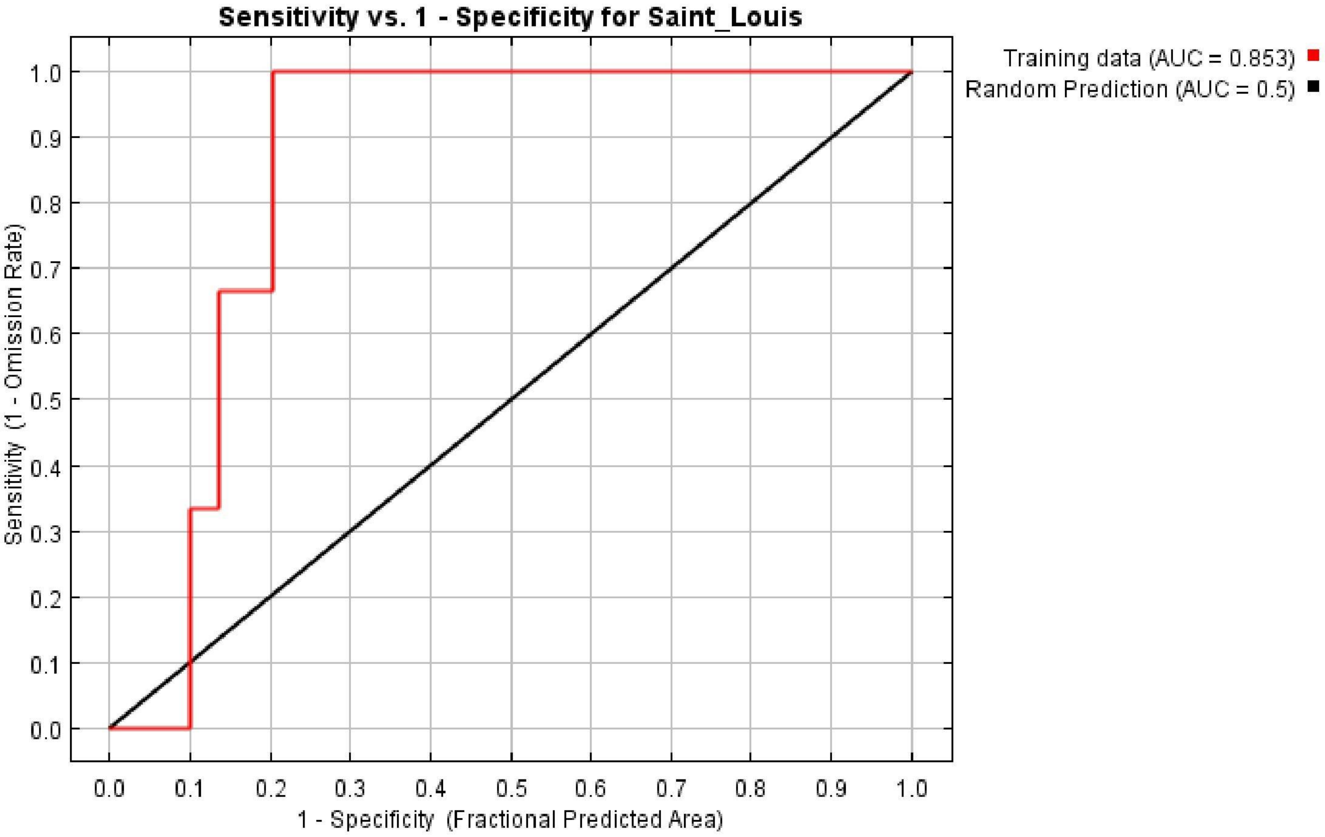

Some common thresholds and corresponding omission rates are as follows. If test data are available, binomial probabilities are calculated exactly if the number of test samples is at most 25, otherwise using a normal approximation to the binomial. These are 1-sided p-values for the null hypothesis that test points are predicted no better than by a random prediction with the same fractional predicted area. The "Balance" threshold minimizes 6 * training omission rate + .04 * cumulative threshold + 1.6 * fractional predicted area.

Cumulative threshold	Logistic threshold	Description	Fractional predicted area	Training omission rate
1.000	0.067	Fixed cumulative value 1	0.885	0.000
5.000	0.148	Fixed cumulative value 5	0.669	0.000
10.000	0.244	Fixed cumulative value 10	0.545	0.000
43.536	0.495	Minimum training presence	0.203	0.000
43.536	0.495	10 percentile training presence	0.203	0.000
43.552	0.495	Equal training sensitivity and specificity	0.203	0.333
43.536	0.495	Maximum training sensitivity plus specificity	0.203	0.000
7.115	0.193	Balance training omission, predicted area and threshold value	0.607	0.000
7.420	0.199	Equate entropy of thresholded and original distributions	0.600	0.000

Pictures of the model

This is a representation of the Maxent model for Saint_Louis. Warmer colors show areas with better predicted conditions. White dots show the presence locations used for training, while violet dots show test locations. Click on the image for a full-size version.

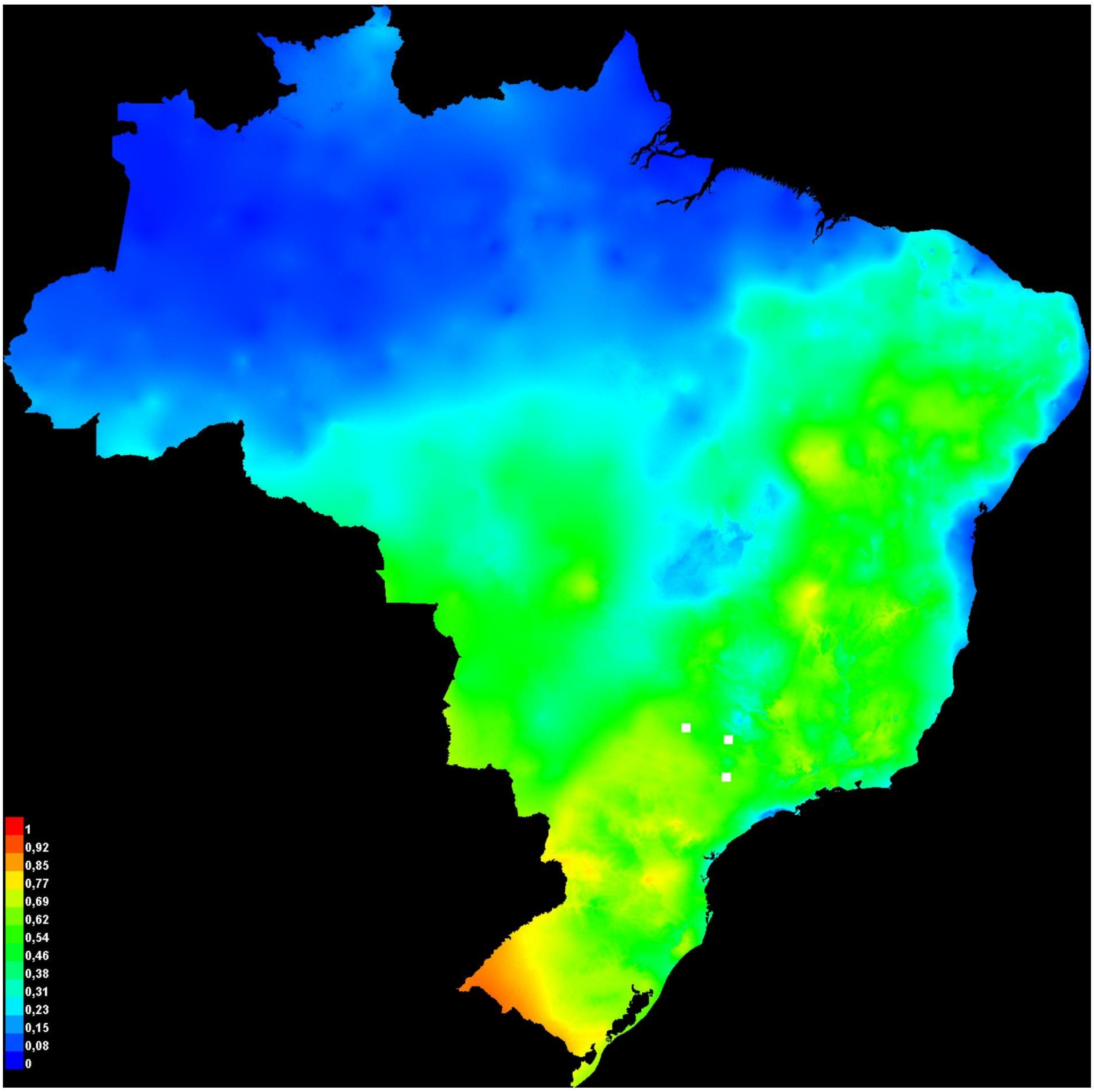

Click here to interactively explore this prediction using the Explain tool. If clicking from your browser does not succeed in starting the tool, try running the script in C:\Users\THIAGO AZEVEDO\Desktop\Elcefalites\Modelos\Modelos_atual_discriminado\Saint_Louis_explain.bat directly. This tool requires the environmental grids to be small enough that they all fit in memory.

Response curves

These curves show how each environmental variable affects the Maxent prediction. The curves show how the logistic prediction changes as each environmental variable is varied, keeping all other environmental variables at their average sample value. Click on a response curve to see a larger version. Note that the curves can be hard to interpret if you have strongly correlated variables, as the model may depend on the

correlations in ways that are not evident in the curves. In other words, the curves show the marginal effect of changing exactly one variable, whereas the model may take advantage of sets of variables changing together.

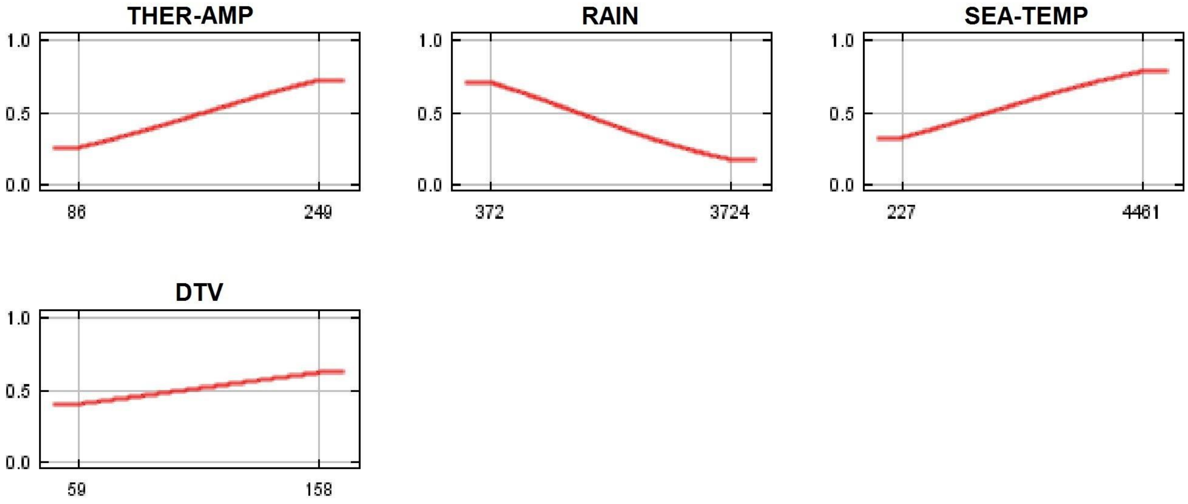

In contrast to the above marginal response curves, each of the following curves represents a different model, namely, a Maxent model created using only the corresponding variable. These plots reflect the dependence of predicted suitability both on the selected variable and on dependencies induced by correlations between the selected variable and other variables. They may be easier to interpret if there are strong correlations between variables.

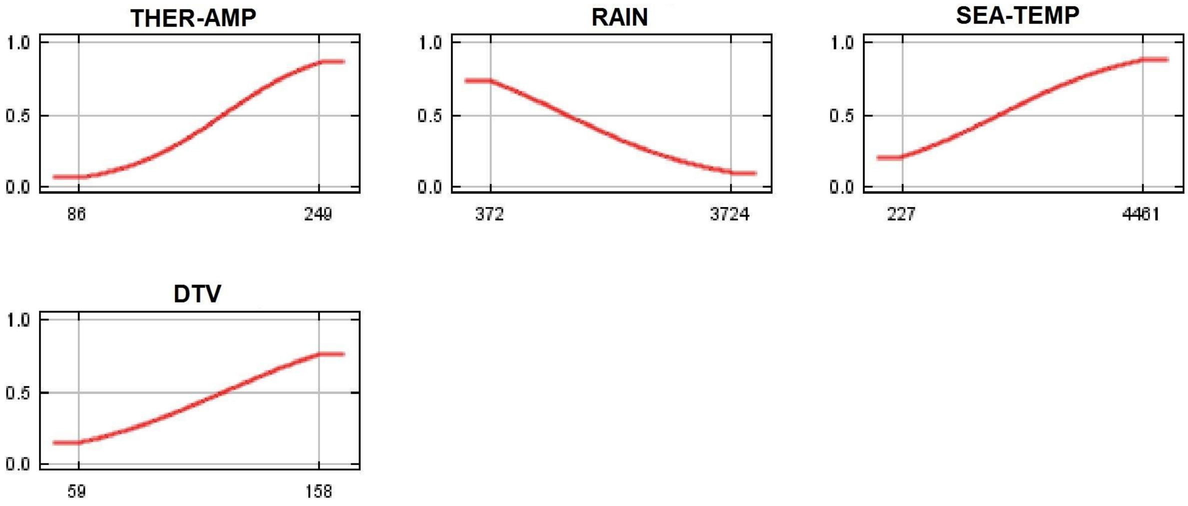

Analysis of variable contributions

The following table gives estimates of relative contributions of the environmental variables to the Maxent model. To determine the first estimate, in each iteration of the training algorithm, the increase in regularized gain is added to the contribution of the corresponding variable, or subtracted from it if the change to the absolute value of lambda is negative. For the second estimate, for each environmental variable in turn, the values of that variable on training presence and background data are randomly permuted. The model is reevaluated on the permuted data, and the resulting drop in training AUC is shown in the table, normalized to percentages. As with the variable jackknife, variable contributions should be interpreted with caution when the predictor variables are correlated.

Variable	Percent contribution	Permutation importance
SEA-TEMP	62.7	97.7
THER-AMP	24.5	0
RAIN	11.5	2.2
DTV	1.4	0.1

Raw data outputs and control parameters

The data used in the above analysis is contained in the next links. Please see the Help button for more information on these.
The model applied to the training environmental layers
The coefficients of the model
The omission and predicted area for varying cumulative and raw thresholds
The prediction strength at the training and (optionally) test presence sites
Results for all species modeled in the same Maxent run, with summary statistics and (optionally) jackknife results

Regularized training gain is 0.511, training AUC is 0.853, unregularized training gain is 0.685.
Algorithm converged after 80 iterations (0 seconds).

The follow settings were used during the run:
3 presence records used for training.
10003 points used to determine the Maxent distribution (background points and presence points).
Environmental layers used (all continuous): amplitermica precipanual sazonalidadetemp vardiurntemp
Regularization values: linear/quadratic/product: 1.000, categorical: 0.605, threshold: 1.970, hinge: 0.500
Feature types used: linear
responsecurves: true
outputdirectory: C:\Users\THIAGO AZEVEDO\Desktop\Elcefalites\Modelos\Modelos_atual_discriminado
samplesfile: C:\Users\THIAGO AZEVEDO\Desktop\Elcefalites\Modelos\Surtos\Saint Louis.csv
environmentallayers: C:\Users\THIAGO AZEVEDO\Desktop\Elcefalites\Raster\dados
Command line used:

Command line to repeat this species model: java density.MaxEnt nowarnings noprefixes -E "" -E Saint_Louis responsecurves "outputdirectory=C:\Users\THIAGO AZEVEDO\Desktop\Elcefalites\Modelos\Modelos_atual_discriminado" "samplesfile=C:\Users\THIAGO AZEVEDO\Desktop\Elcefalites\Modelos\Surtos\Saint Louis.csv" "environmentallayers=C:\Users\THIAGO AZEVEDO\Desktop\Elcefalites\Raster\dados" -N altitude -N tempanual

Maxent model for Rocio

This page contains some analysis of the Maxent model for Rocio, created Fri Nov 11 01:09:59 BRST 2016 using Maxent version 3.3.3k. If you would like to do further analyses, the raw data used here is linked to at the end of this page.

Analysis of omission/commission

The following picture shows the omission rate and predicted area as a function of the cumulative threshold. The omission rate is is calculated both on the training presence records, and (if test data are used) on the test records. The omission rate should be close to the predicted omission, because of the definition of the cumulative threshold.

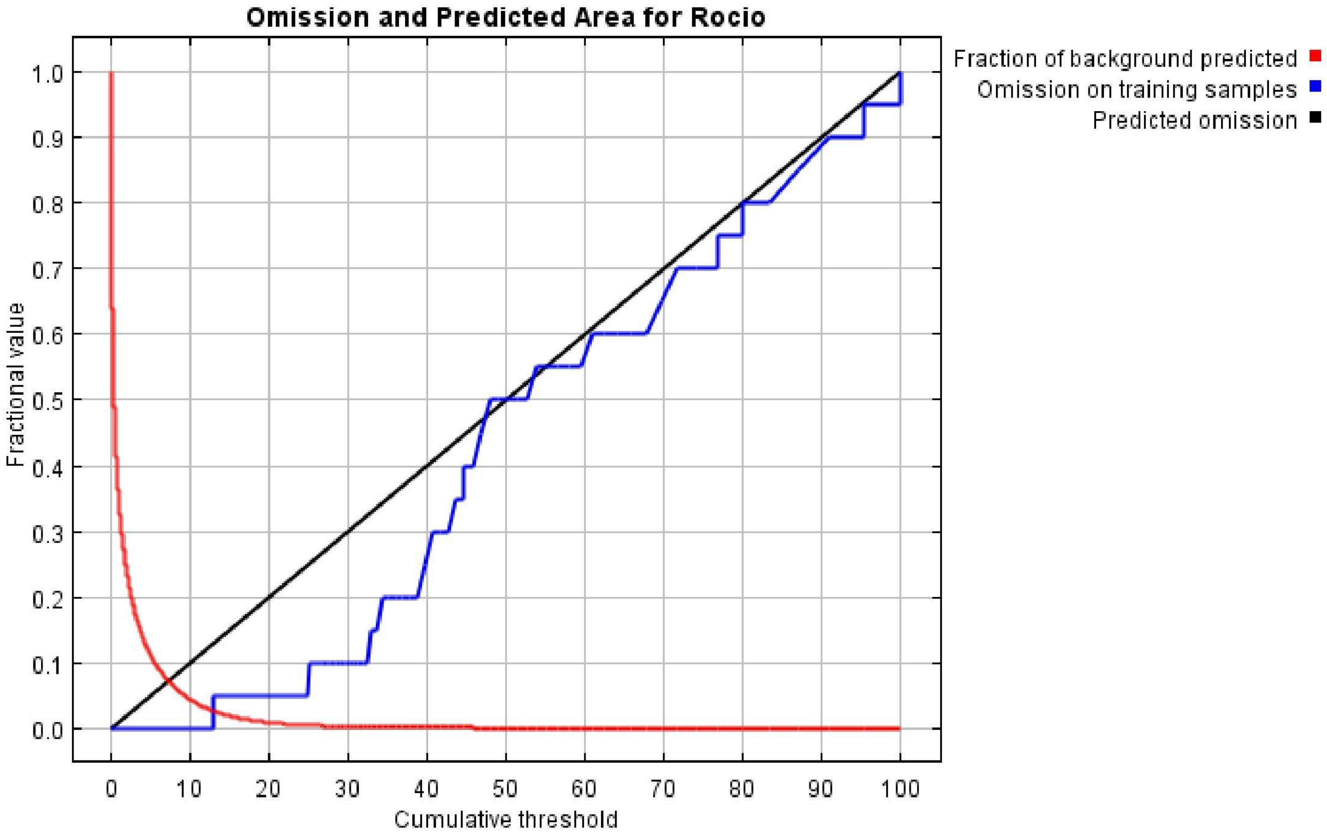

The next picture is the receiver operating characteristic (ROC) curve for the same data. Note that the specificity is defined using predicted area, rather than true commission (see the paper by Phillips, Anderson and Schapire cited on the help page for discussion of what this means). This implies that the maximum achievable AUC is less than 1. If test data is drawn from the Maxent distribution itself, then the maximum possible test AUC would be 0.973 rather than 1; in practice the test AUC may exceed this bound.

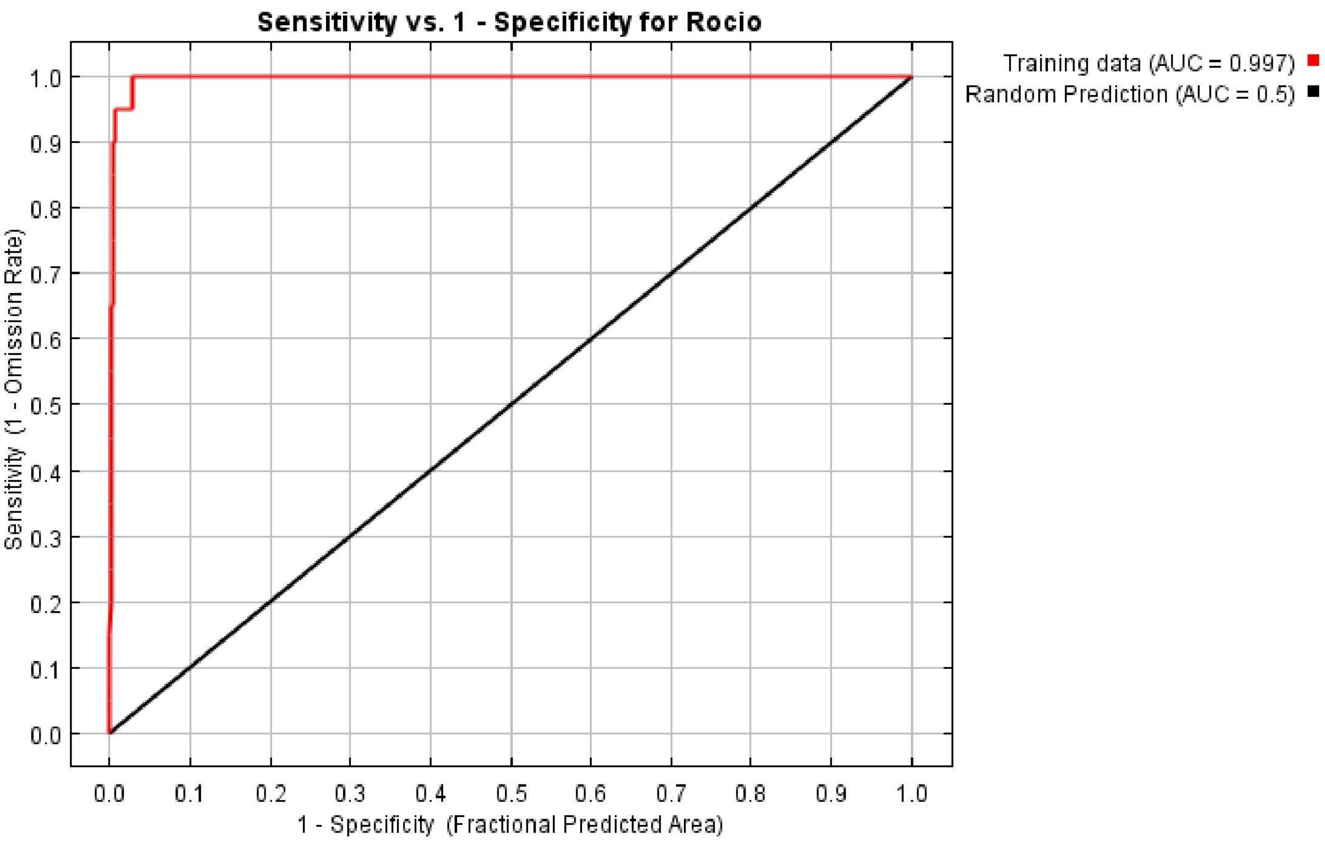

Some common thresholds and corresponding omission rates are as follows. If test data are available, binomial probabilities are calculated exactly if the number of test samples is at most 25, otherwise using a normal approximation to the binomial. These are 1-sided p-values for the null hypothesis that test points are predicted no better than by a random prediction with the same fractional predicted area. The "Balance" threshold minimizes 6 * training omission rate + .04 * cumulative threshold + 1.6 * fractional predicted area.

Cumulative threshold	Logistic threshold	Description	Fractional predicted area	Training omission rate
1.000	0.001	Fixed cumulative value 1	0.341	0.000
5.000	0.008	Fixed cumulative value 5	0.112	0.000
10.000	0.024	Fixed cumulative value 10	0.045	0.000
12.926	0.041	Minimum training presence	0.028	0.000
32.418	0.526	10 percentile training presence	0.004	0.100
12.948	0.041	Equal training sensitivity and specificity	0.028	0.050
12.926	0.041	Maximum training sensitivity plus specificity	0.028	0.000
4.715	0.008	Balance training omission, predicted area and threshold value	0.119	0.000
15.518	0.062	Equate entropy of thresholded and original distributions	0.019	0.050

Pictures of the model

This is a representation of the Maxent model for Rocio. Warmer colors show areas with better predicted conditions. White dots show the presence locations used for training, while violet dots show test locations. Click on the image for a full-size version.

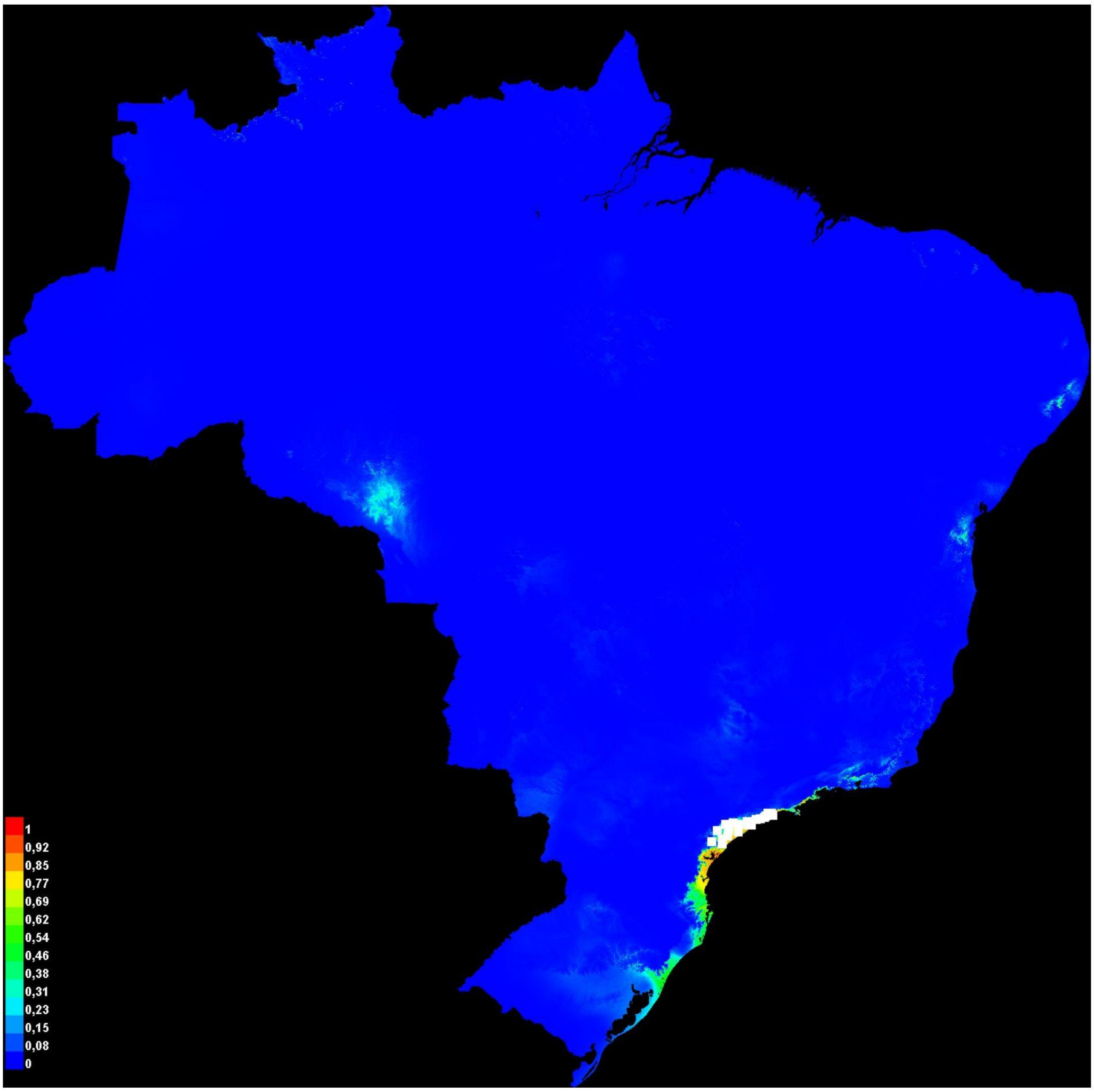

Click here to interactively explore this prediction using the Explain tool. If clicking from your browser does not succeed in starting the tool, try running the script in C:\Users\THIAGO AZEVEDO\Desktop\Elcefalites\Modelos\Modelos_atual_discriminado\Rocio_explain.bat directly. This tool requires the environmental grids to be small enough that they all fit in memory.

Response curves

These curves show how each environmental variable affects the Maxent prediction. The curves show how the logistic prediction changes as each environmental variable is varied, keeping all other environmental variables at their average sample value. Click on a response curve to see a larger version. Note that the curves can be hard to interpret if you have strongly correlated variables, as the model may depend on the

correlations in ways that are not evident in the curves. In other words, the curves show the marginal effect of changing exactly one variable, whereas the model may take advantage of sets of variables changing together.

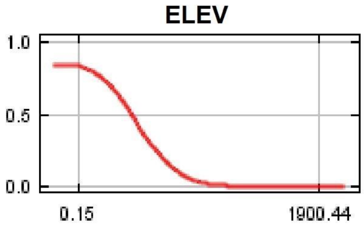

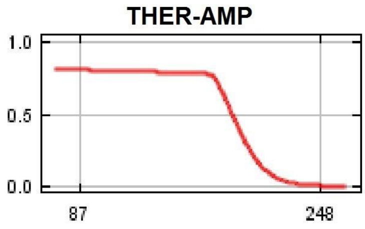

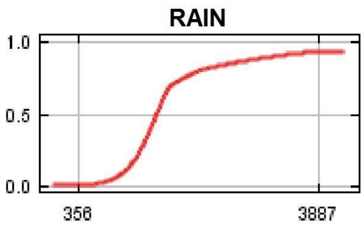

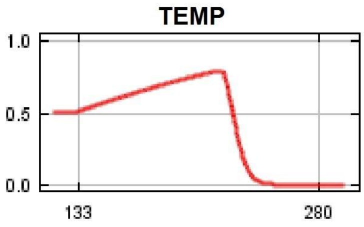

In contrast to the above marginal response curves, each of the following curves represents a different model, namely, a Maxent model created using only the corresponding variable. These plots reflect the dependence of predicted suitability both on the selected variable and on dependencies induced by correlations between the selected variable and other variables. They may be easier to interpret if there are strong correlations between variables.

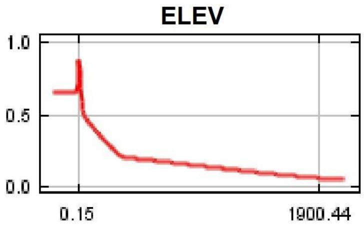

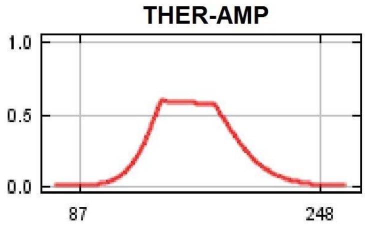

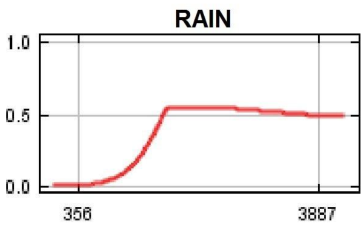

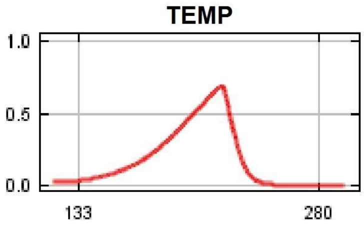

Analysis of variable contributions

The following table gives estimates of relative contributions of the environmental variables to the Maxent model. To determine the first estimate, in each iteration of the training algorithm, the increase in regularized gain is added to the contribution of the corresponding variable, or subtracted from it if the change to the absolute value of lambda is negative. For the second estimate, for each environmental variable in turn, the values of that variable on training presence and background data are randomly permuted. The model is reevaluated on the permuted data, and the resulting drop in training AUC is shown in the table, normalized to percentages. As with the variable jackknife, variable contributions should be interpreted with caution when the predictor variables are correlated.

Variable	Percent contribution	Permutation importance
TEMP	50.7	85.6
ELEV	25.8	6.1
RAIN	13.6	5.5
THER-AMP	9.9	2.8

Raw data outputs and control parameters

The data used in the above analysis is contained in the next links. Please see the Help button for more information on these.
The model applied to the training environmental layers
The coefficients of the model
The omission and predicted area for varying cumulative and raw thresholds
The prediction strength at the training and (optionally) test presence sites
Results for all species modeled in the same Maxent run, with summary statistics and (optionally) jackknife results

Regularized training gain is 3.960, training AUC is 0.997, unregularized training gain is 4.586.
Algorithm converged after 360 iterations (2 seconds).

The follow settings were used during the run:
20 presence records used for training.
10020 points used to determine the Maxent distribution (background points and presence points).
Environmental layers used (all continuous): altitude amplitermica precipanual tempanual
Regularization values: linear/quadratic/product: 0.442, categorical: 0.250, threshold: 1.800, hinge: 0.500
Feature types used: linear quadratic hinge
responsecurves: true
outputdirectory: C:\Users\THIAGO AZEVEDO\Desktop\Elcefalites\Modelos\Modelos_atual_discriminado
samplesfile: C:\Users\THIAGO AZEVEDO\Desktop\Elcefalites\Modelos\Surtos\Rocio.csv
environmentallayers: C:\Users\THIAGO AZEVEDO\Desktop\Elcefalites\Raster\dados
Command line used:

Command line to repeat this species model: java density.MaxEnt nowarnings noprefixes -E "" -E Rocio responsecurves "outputdirectory=C:\Users\THIAGO AZEVEDO\Desktop\Elcefalites\Modelos\Modelos_atual_discriminado" "samplesfile=C:\Users\THIAGO AZEVEDO\Desktop\Elcefalites\Modelos\Surtos\Rocio.csv" "environmentallayers=C:\Users\THIAGO AZEVEDO\Desktop\Elcefalites\Raster\dados" -N sazonalidadetemp -N vardiurntemp
